# Trends in Pediatric Blood Pressure–Lowering Prescription Fills During 2017–2023

**DOI:** 10.1016/j.focus.2025.100356

**Published:** 2025-04-22

**Authors:** Ashutosh Kumar, Nicole L. Therrien, John Ogwuegbu, Siran He, Jun Soo Lee, Omoye Imoisili, Elizabeth A. Lundeen, Katrice Lampley, Sandra L. Jackson

**Affiliations:** 1 Division for Heart Disease and Stroke Prevention, National Center for Chronic Disease Prevention and Health Promotion, Centers for Disease Control and Prevention, Atlanta, Georgia; 2 The Bizzell Group, New Carrollton, Maryland; 3 Oak Ridge Institute for Science and Education, Oak Ridge, Tennessee; 4 ASRT, Inc, Atlanta, Georgia

**Keywords:** Blood pressure–lowering medications, pediatric hypertension, blood pressure, clinical practice guideline, antihypertensives

## Abstract

**Introduction:**

There are no national estimates for blood pressure–lowering prescription trends among the U.S. pediatric population. This study describes trends in blood pressure–lowering prescription fills among individuals aged 3–17 years by sex and age group.

**Methods:**

Data were obtained from IQVIA’s Total Patient Tracker database covering 94% of all outpatient retail prescription fills in the U.S. The key outcome was blood pressure–lowering prescription fills during 2017–2023, utilizing a list of 113 generic medications from 21 drug classes. In addition, a subset of 20 medications recommended in the 2017 American Academy of Pediatrics guideline was examined. Annual population percentage and percentage change compared with 2017 were reported, and average annual percentage change was estimated using Joinpoint regression.

**Results:**

From 2017 to 2023, blood pressure–lowering prescription fills among those aged 3–17 years increased slightly from 1.93% (95% CI=1.88%, 1.98%) to 2.09% (95% CI=2.04%, 2.14%). Among males, blood pressure–lowering prescription fills remained stable (between 2.32% and 2.38%; average annual percentage change= −0.3%; *p*=0.545), whereas fills among females increased by 23.9% (from 1.49% to 1.84%; average annual percentage change=4.16%; *p*<0.001). The sharpest increase occurred among females aged 13–17 years (from 2.26% to 3.17%; average annual percentage change=6.3%; *p*<0.001). Prescription fills for guideline-recommended medications either remained stable or declined, with some variation by sex and age group.

**Conclusions:**

Results indicate growth in blood pressure–lowering prescription fills, especially among females aged 13–17 years. Increases were driven by medications not included in the 2017 American Academy of Pediatrics guideline, suggesting that blood pressure–lowering medications may be increasingly prescribed for conditions other than pediatric hypertension.

## Highlights

• Data on blood pressure–lowering prescription fills among U.S. children are scarce.
• From 2017 to 2023, fills among those aged 3–17 years increased from 1.9% to 2.1%.
• Fills remained stable among males and increased among females.
• Females aged 13–17 years experienced the sharpest increase (40.3%).
• Increases were driven by medications not included in the 2017 American Academy of Pediatrics guideline.

## INTRODUCTION

Hypertension, a major modifiable risk factor for cardiovascular morbidity and mortality, affects nearly 4% of U.S. children and adolescents aged 8–17 years.^1^^,^^2^ Pediatric hypertension is associated with left ventricular hypertrophy and atherosclerosis in youth, which may lead to an increased risk of hypertension during adulthood as well as related cardiovascular events, end-stage renal disease, and premature mortality.^3^, ^4^, ^5^, ^6^, ^7^, ^8^, ^9^ However, pediatric hypertension frequently goes undiagnosed,^10^, ^11^, ^12^ leading to missed opportunities for early detection and treatment.

In 2017, the American Academy of Pediatrics (AAP) changed its Clinical Practice Guideline^13^ for the classification of pediatric hypertension, introducing new normative pediatric blood pressure (BP) tables that lowered BP thresholds among children aged 0–12 years and recommended a cut point of 130/80 mm Hg for adolescents aged ≥13 years.^14^ As a result, the prevalence of hypertension among individuals aged 8–17 years increased from 1.9%^15^ to 3.5%.^2^^,^^13^^,^^16^ For pediatric hypertension, lifestyle interventions such as a healthy diet, regular exercise, and weight reduction are recommended as the first line of treatment, followed by pharmacologic therapy.^17^ The 2017 AAP guideline may have led to increased recognition and diagnosis of pediatric hypertension,^2^^,^^18^^,^^19^ which could have resulted in increased use of BP-lowering medications.^20^ Moreover, the pharmacologic treatment of pediatric hypertension may be on the rise because the number of U.S. Food and Drug Administration (FDA)–approved medications for the treatment of pediatric hypertension increased from 0 in 2000 to 11 in 2014.^21^^,^^22^ In addition, BP-lowering medications may also be prescribed for other conditions, such as sleep disorder,^23^ anxiety,^24^^,^^25^ autism spectrum disorder (ASD),^26^ and polycystic ovary syndrome (PCOS).^27^

More nuanced information about BP-lowering medications among the pediatric population could help inform clinicians, practitioners, and researchers about current BP-lowering drug-prescribing practices in the context of 3 factors: the updated 2017 AAP guideline,^13^ the recent rise in pharmacologic treatment options that have been studied in pediatric hypertension populations, and the potential use of BP-lowering medications for other indications.^21^^,^^28^^,^^29^ The primary objective of this study was to present recent temporal trends from 2017 to 2023 for BP-lowering prescription fills among U.S. individuals aged 3–17 years by sex and age group, leveraging a national prescription data set. The secondary objective was to assess these trends for the subset of BP-lowering medications that have been recommended for outpatient management of chronic hypertension in the 2017 AAP guideline.^13^

## METHODS

### Population

The prescription fills of BP-lowering medications for U.S. individuals aged 3–17 years were obtained from IQVIA’s Total Patient Tracker (TPT) prescription data set. The TPT database uses statistical weights to provide deduplicated annual counts of nationally projected numbers of individuals who filled any outpatient retail prescription for BP-lowering medications. With 94% coverage of all outpatient retail prescription fills in the U.S., the TPT database collects prescription information directly from the payers and retail pharmacies, including pharmacy chains, independent pharmacies, food stores, and mass merchandisers,^30^ and has been used for regulatory monitoring, public health surveillance, and analyses of trends.^31^ Fills prescribed by veterinarians, dentists, and naturopaths were excluded because BP-lowering medications would be less commonly used in these settings.

### Measures

Separately, IQVIA’s National Prescription Audit data were used to identify generic and branded medications. For the primary objective of assessing trends in BP-lowering prescription fills, 21 drug classes for 113 generic products were included (Appendix Table 1, available online), utilizing the most granular level of IQVIA’s Uniform System of Classification 5 Codes 31100–31900 and 41110–41190.^32^ For the secondary objective, trends in prescription fills were assessed for medications included in Table 17 of the 2017 AAP guideline for outpatient management of chronic hypertension among the pediatric population, referred to as guideline-recommended BP-lowering medications in the remaining parts of this paper.^13^ There were 20 drug products from 4 drug classes that were classified as guideline-recommended BP-lowering medications (Appendix Table 1, available online). Annual counts and population percentages of prescription fills for BP-lowering medications and for guideline-recommended medications for all years were reported (Appendix Tables 2 and 3, available online). Furthermore, trends by individual BP-lowering medications were presented for each drug class, stratified by age groups (Appendix Table 4, available online). Among BP-lowering medications, the trends for medications that increased in prescription fills by more than 500 individuals during 2017–2023 were reported (Appendix Table 5, available online). This secondary data analysis of deidentified data was considered not to be human subjects research and was exempt from IRB approval.

### Statistical Analysis

Annual deduplicated counts of individuals who filled any BP-lowering medication each year from 2017 to 2023 were provided. Results for 9 groups overall and for stratified subgroups were reported: total, males, females, males aged 3–7 years, males aged 8–12 years, males aged 13–17 years, females aged 3–7 years, females aged 8–12 years, and females aged 13–17 years. U.S. population totals from the CDC WONDER data set^33^ for each subgroup were used to report the prevalence of BP-lowering prescription fills as population percentages. IQVIA’s TPT data were used to obtain 95% CIs for the population percentages of BP-lowering prescription fills. Using population percentages, absolute (nonregression-based) percentage changes in individuals with BP-lowering prescription fills were reported compared with 2017, and trend changes, overall and stratified by sex and age group (Table 1, Table 2). Because population data by single-year age for 2023 were unavailable, the 2022 population data were used for 2023. The results for generic medications were reported because these represented the vast majority of prescription fills.

**Table 1:** Annual Total Number and Population Percentage of Unique Individuals Aged 3–17 Years With Prescription Fills for Generic and Guideline-Recommended BP-Lowering Medications Overall and by Sex and Age groups, IQVIA TPT, 2017–2023^a^

	2017				2023				
Characteristics	Count	Population percentage^c^	Low CI	High CI	Count	Population percentage^c^	Low CI	High CI	Change^b^ (%)
**BP-lowering medications**									
Total^d^	1,190,800	1.93%	1.88%	1.98%	1,284,456	2.09%	2.04%	2.14%	8.27%*
Male^d^	749,932	2.38%	2.31%	2.45%	730,257	2.32%	2.26%	2.38%	−2.47%
Female^d^	449,489	1.49%	1.44%	1.54%	553,516	1.84%	1.79%	1.90%	23.90%*
Male: 3–7 years	153,123	1.49%	1.42%	1.56%	154,670	1.55%	1.48%	1.62%	3.90%*
Male: 8–12 years	343,202	3.25%	3.13%	3.36%	311,676	3.00%	2.89%	3.11%	−7.62%*
Male: 13–17 years	276,212	2.59%	2.49%	2.68%	286,716	2.58%	2.49%	2.68%	−0.09%
Female: 3–7 years	69,268	0.71%	0.67%	0.74%	69,006	0.72%	0.68%	0.76%	2.47%
Female: 8–12 years	158,212	1.56%	1.49%	1.63%	159,870	1.61%	1.54%	1.69%	3.46%
Female: 13–17 years	231,156	2.26%	2.17%	2.35%	334,579	3.17%	3.05%	3.28%	40.29%*
**2017 AAP guideline–recommended BP-lowering medications**									
Total^d^	120,838	0.20%	0.19%	0.21%	100,948	0.16%	0.16%	0.17%	−16.14%
Male^d^	70,743	0.22%	0.21%	0.24%	59,226	0.19%	0.18%	0.20%	−16.15%
Female^d^	51,861	0.17%	0.16%	0.18%	41,119	0.14%	0.13%	0.15%	−20.23%*
Male: 3–7 years	7,386	0.07%	0.06%	0.08%	6,527	0.07%	0.06%	0.07%	−9.10%
Male: 8–12 years	19,296	0.18%	0.17%	0.20%	15,557	0.15%	0.14%	0.16%	−17.98%*
Male: 13–17 years	43,749	0.41%	0.38%	0.44%	38,136	0.34%	0.32%	0.37%	−16.10%
Female: 3–7 years	5,799	0.06%	0.05%	0.07%	4,944	0.05%	0.05%	0.06%	−12.32%
Female: 8–12 years	14,704	0.15%	0.13%	0.16%	11,367	0.11%	0.10%	0.13%	−20.85%
Female: 13–17 years	31,036	0.30%	0.28%	0.33%	25,474	0.24%	0.22%	0.26%	−20.44%*

*Note:* Boldface indicates statistical significance (*p≤0.05).

a This table presents the trend of prescription fills for BP-lowering generic medications and for medications mentioned in Table 17 of the 2017 AAP guideline among children and adolescents aged 3–17 years from IQVIA’s TPT database. Drug prescriptions for unspecified sex and age groups were excluded.

b Statistically significant values at *p*≤0.05 for change in population percentage in 2023 compared to 2017 are shown.

c Population percentages are shown with 95% CI utilizing CIs obtained from TPT database.

d The aggregate counts of sex and age groups were generated separately for age groups 3–17 years, excluding results for unspecified sex and age groups. The total aggregate numbers are lower than the sum of numbers from subgroups because the deduplication process removes multiple entries for the same person appearing in different age bands while removing all conflicting/missing reports of sex and age groups. AAP, American Academy of Pediatrics; BP, blood pressure; TPT, Total Patient Tracker.

**Table 2:** Joinpoint Results for Generic BP-Lowering Prescription Fills in Population Percentage Overall and by Sex and Age Group Among Individuals Aged 3–17 Years, IQVIA Total Patient Tracker, 2017–2023^a^

	Model details		AAPC			
Group	Join points	Joinpoint year	AAPC	95% lower CI	95% higher CI	*p*-value
BP-lowering medications^b^						
Total	0	NA	1.48*	0.52	2.45	0.011
Male	1	2021	−0.30	−1.28	0.68	0.545
Female	0	NA	4.16*	2.91	5.42	<0.001
Male 3–7 years	1	2021	0.72*	0.15	1.29	0.013
Male 8–12 years	1	2020	−1.36*	−2.32	−0.39	0.016
Male 13–17 years	0	NA	−0.08	−0.88	0.74	0.081
Female: 3–7 years	1	2019	0.30	−0.42	1.03	0.411
Female: 8–12 years	1	2019	0.71	−0.28	1.70	0.162
Female: 13–17 years	0	NA	6.30*	5.00	7.62	<0.001
2017 AAP guideline–recommended BP-lowering medications^c^						
Total	1	2020	−2.70	−6.32	1.07	0.158
Male	1	2020	−2.60	−5.91	0.82	0.135
Female	1	2020	−3.43*	−6.71	−0.03	0.047
Male 3–7 years	1	2020	−0.37	−7.57	7.39	0.922
Male 8–12 years	1	2020	−3.17*	−5.84	−0.42	0.024
Male 13–17 years	1	2020	−2.83	−5.69	0.12	0.061
Female: 3–7 years	1	2019	−1.85	−11.42	8.76	0.722
Female: 8–12 years	1	2020	−3.80	−7.48	0.01	0.051
Female: 13–17 years	1	2020	−3.82*	−6.79	−0.74	0.015

*Note:* Boldface indicates statistical significance (*p≤0.05).

a This table shows the Joinpoint regression results for population percentage with BP-lowering prescription fills and guideline-recommended BP-lowering prescription fills in AAPC for all groups for Years 2017–2023. All AAPC estimates are shown with a 95% CI.

b Population estimates with 95% CIs, percentage change from 2017 to 2023, and *p*-value from linear trend are presented in Appendix Table 2 (available online).

c Population estimates with 95% CIs, percentage change from 2017 to 2023, and *p*-value from linear trend are presented in Appendix Table 3 (available online). AAP, American Academy of Pediatrics; AAPC, average annual percentage change; NA, not applicable.

Joinpoint regression (JoinPoint 5.0.2, National Cancer Institute, Bethesda, MD) with the permutation-based method was used to determine statistically significant trends from 2017 to 2023.^34^ The population percentage with BP-lowering prescription fills was selected as the outcome in the log model with uncorrelated errors for Joinpoint regression. Average annual percentage change (AAPC) for the prevalence of BP-lowering prescription fills during 2017–2023 was reported from the Joinpoint regression (Table 2). Population percentages and Joinpoint regression results were reported with 95% CIs, and *p*<0.05 for AAPC was used to report statistically significant trend.

## RESULTS

**Figure 1:**
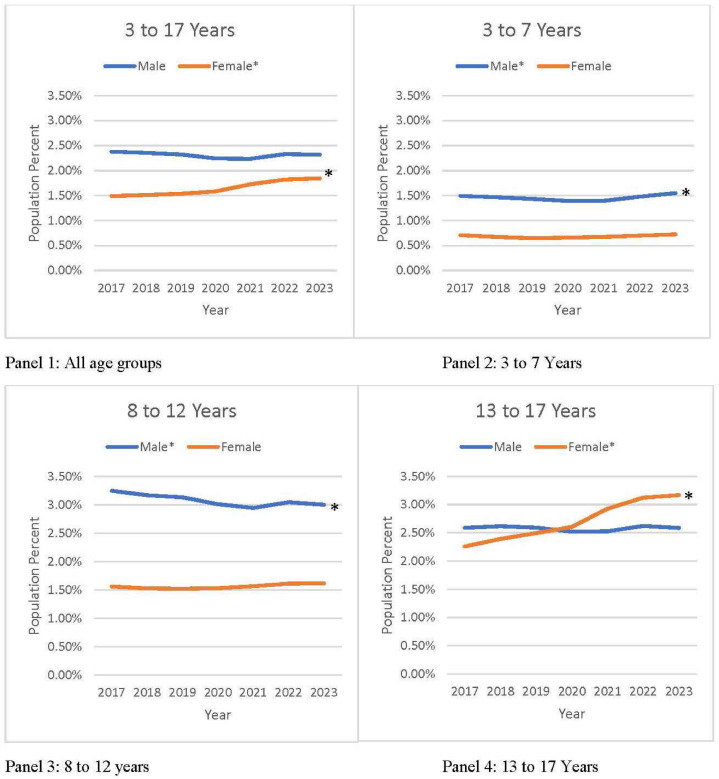
Percentage of U.S. population aged 3–17 years with prescription fills for generic BP-lowering medications by sex and age group, IQVIA Total Patient Tracker, 2017–2023. This figure shows the annual trend of individuals with prescription fills for generic BP-lowering medication among males and females aged 3–17 years during 2017–2023 using U.S. population estimates by sex and age group for all years. Owing to the unavailability of data for 2023, the population total of Year 2022 was also used for Year 2023. Population percentages with 95% CIs and changes from 2017 to 2023 are presented in Appendix Table 2. Trends with *p*-value are presented. BP, blood pressure.

From 2017 to 2023, approximately 2% of the U.S. population aged 3–17 years filled prescriptions for BP-lowering medications, increasing slightly from 1.19 million or 1.93% (95% CI=1.88%, 1.98%) in 2017 to 1.28 million or 2.09% (95% CI=2.04%, 2.14%) in 2023, which was an 8.27% increase (Figure 1 and Table 1, Table 2). Compared with 2017, there was a continuous rise in percentage of individuals with BP-lowering prescription fills in all years except 2020 with an AAPC for 2017–2023 of 1.48% (95% CI=0.52%, 2.45%, *p*=0.011) (Appendix Table 2, available online). However, trends differed for males and females. Among males aged 3–17 years, BP-lowering prescription fills remained stable between 2.32% and 2.38% (*p*=0.545). Among females aged 3–17 years, the percentage of individuals with BP-lowering prescription fills increased from 1.49% (95% CI=1.44%, 1.54%) (0.45 million) in 2017 to 1.84% (95% CI=1.79%, 1.90%) (0.55 million) in 2023 (a 23.9% increase; AAPC=4.16% [95% CI=2.91%, 5.42%, *p*<0.001]).

Among males aged 3–7 years, BP-lowering prescription fills increased slightly (by 3.9%) from 1.49% in 2017 to 1.55% in 2023 (AAPC=0.72% [95% CI=0.15%, 1.29%], *p*=0.013) (Figure 1 and Table 1, Table 2). Among males aged 8–12 years, BP-lowering prescription fills declined from 0.34 million or 3.25% (95% CI=3.13%, 3.36%) in 2017 to 0.31 million or 3.00% (95% CI=2.89%, 3.11%) in 2023 (a decline of 7.62%; AAPC= −1.36% [95% CI= −2.32%, −0.39%, *p*=0.016]). However, BP-lowering prescription fills remained stable between 2.58% and 2.59% (*p*=0.081) for those aged 13–17 years.

Among females, BP-lowering prescription fills remained stable for those aged 3–7 years (between 0.71% and 0.72%, *p*=0.411) and for those aged 8–12 years (between 1.56% and 1.61%, *p*=0.162) during 2017–2023 (Figure 1 and Table 1, Table 2). However, females aged 13–17 years experienced a sharp increase in BP-lowering prescription fills by 40.29%: from 0.23 million or 2.26% (95% CI=2.17%, 2.35%) in 2017 to 0.33 million or 3.17% (95% CI=3.05%, 3.28%) in 2023. Compared with 2017 fills, females aged 13–17 years experienced a continuous increase in BP-lowering prescription fills in all years until 2023 (AAPC=6.30% [95% CI=5.00%, 7.62%, *p*<0.001]).

**Figure 2:**
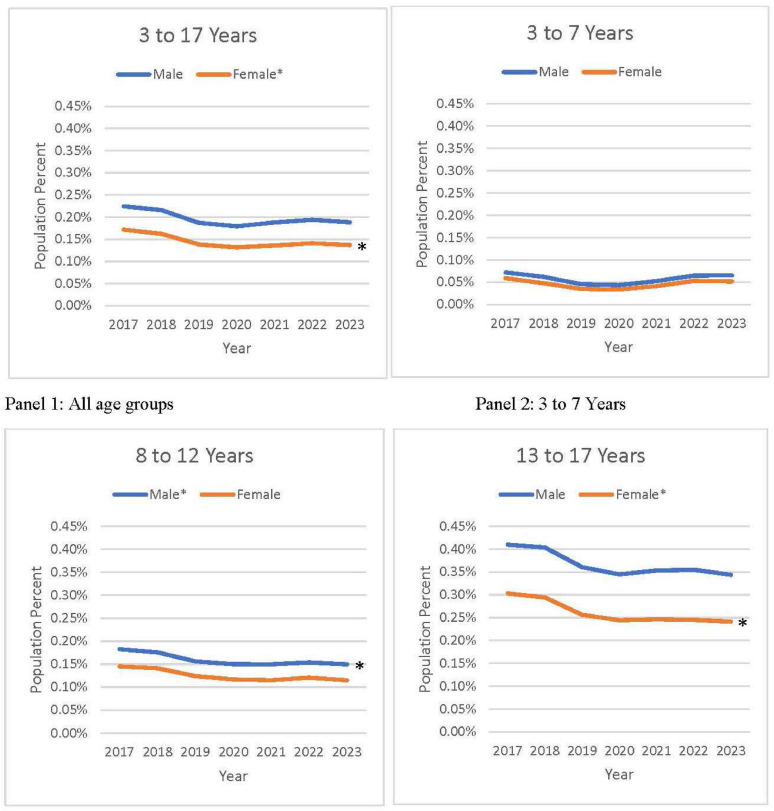
Percentage of U.S. population aged 3–17 years with prescription fills for generic BP-lowering medications mentioned in the 2017 AAP guideline for outpatient management of chronic hypertension by sex and age group, IQVIA Total Patient Tracker, 2017–2023. This figure shows the annual prescription trend of generic BP-lowering medications mentioned in the 2017 AAP guideline among males and females aged 3–17 years during 2017–2023 using U.S. population estimates by sex and age group for all years. Owing to the unavailability of data for 2023, the population total of Year 2022 was also used for Year 2023. Population percentages with 95% CIs and changes from 2017 to 2023 are presented in Appendix Table 3. Trends with *p*-value are presented. AAP, American Academy of Pediatrics; BP, blood pressure.

Prescription fills for guideline-recommended BP-lowering medications were a small fraction (approximately 10.14% in 2017 to 7.85% in 2023) of all BP-lowering medications during this period. Prescription fills for guideline-recommended medications were higher among males than among females for all age groups across all years (Figure 2 and Table 1, Table 2). During 2017–2023, prescription fills for guideline-recommended medications among individuals aged 3–17 years remained stable between 0.16% and 0.20% overall (*p*=0.158) and between 0.19% and 0.22% for males (*p*=0.135), and declined for females: from 51,861 or 0.17% (95% CI=0.16%, 0.18%) in 2017 to 41,119 or 0.14% (95% CI=0.13%, 0.15%) in 2023 (a decline of 20.23%; AAPC= −3.43% [95% CI= −6.71%, −0.03%, *p*=0.047]) (Appendix Table 3, available online). Prescription fills for guideline-recommended medications remained stable for most sex–age groups, although they declined among males aged 8–12 years (from 0.18% to 0.15%; AAPC= −3.17% [95% CI= −5.84%, −0.42%], *p*=0.024) and females aged 13–17 years (from 0.30% to 0.24%; AAPC= −3.82% [95% CI= −6.79%, −0.74%], *p*=0.015) during 2017–2023.

Annual trends in total number of unique individuals with prescription fills for 113 BP-lowering medications are presented in Appendix Table 4 (available online). There were 10 BP-lowering medications with an increase of more than 500 in the number of unique individuals with prescription fills in 2023 compared with those in 2017 (Appendix Table 5, available online), and the 4 medications with the greatest increase (>10,000) were spironolactone, clonidine, propranolol, and prazosin.

## DISCUSSION

To authors’ knowledge, this study is the first to report nationwide trends in BP-lowering prescription fills among U.S. children and adolescents aged 3–17 years. A comprehensive data set covering 94% of outpatient retail prescription fills was used to assess the trend of prescription fills for BP-lowering medications among the U.S. pediatric population during 2017–2023. Three key findings were reported. First, approximately 2% of U.S. children and adolescents filled BP-lowering prescriptions during 2017–2023. Second, although there was little change in prescription fills among males, there was an increase among females (absolute change=23.90%), with the sharpest increase among females aged 13–17 years (absolute change=40.29%). Third, prescription fills for guideline-recommended medications^13^ represented only a small fraction (about 8%–10%) of all BP-lowering prescription fills, and the prescription fills for guideline-recommended medications remained stable overall with some variation by sex and age groups during 2017–2023.

This study complements prior studies that estimated the prevalence of hypertension among U.S. children and adolescents. Hardy et al.^4^^,^^35^ reported age-adjusted prevalence of hypertension increasing among adolescents aged 13–17 years (from 2.5% to 3.7%) from 2011–2014 to 2015–2018. The results of this study align with these findings, with an increase in BP-lowering prescription fills among females aged 13–17 years during 2017–2023. This trend suggests the growth of pharmacologic treatment of pediatric hypertension or rising prevalence of pediatric hypertension and other conditions such as anxiety, ASD, and PCOS, which may include BP-lowering medications as one of the treatment strategies.^36^

BP-lowering medications are recommended for children and adolescents with hypertension after unsuccessful nonpharmacologic therapy, those with symptomatic hypertension or Stage 2 hypertension, or those with hypertension associated with chronic kidney disease or diabetes mellitus therapy.^13^^,^^17^ Pharmacologic treatment of pediatric hypertension is often based on the existence of comorbidities.^17^ For example, angiotensin-converting enzyme inhibitors or angiotensin receptor blockers are prescribed among children depending on the underlying mechanisms leading to BP elevation, including obesity-related comorbidities. Similarly, angiotensin-converting enzyme inhibitors combined with beta-blockers and diuretics are recommended for individuals with heart failure or reduced left ventricular ejection fraction.^37^ Recent studies reveal that newly classified youth with hypertension, on the basis of the 2017 AAP guideline, were more likely to have risk factors such as obesity, adverse lipid profiles, and prediabetes.^16^ Nugent and colleagues^38^ reported elevated or hypertensive BP among nearly 1 in 3 children aged 3–12 years with overweight or obesity. There has been an increasing incidence of other risk factors—obesity,^39^^,^^40^ Type 1 diabetes, and Type 2 diabetes^41^—among youth in the U.S. Therefore, an increasing trend of prescription fills for BP-lowering medications might suggest a rising occurrence of complex cardiometabolic conditions, either hypertension related or otherwise, among U.S. children and adolescents. More research is needed to understand these observed patterns.

This study documented sex-specific differences in trends of BP-lowering prescription fills, with a slight decline among males and a notable increase among females during 2017–2023. Prior studies, utilizing data mostly from years before the 2017 guideline, reveal sex-specific differences in pediatric hypertension that could explain an increasing trend of BP-lowering medications among females. For example, Hardy et al.^4^ reported a slight increase in hypertension prevalence among females in both age groups (ages 8–12 and 13–17 years) in contrast to a decline in hypertension prevalence among males aged 8–12 years from 2011–2014 to 2015–2018. Childhood obesity is a risk factor for hypertension, and some recent estimates suggest sharper increases in the prevalence of obesity or severe obesity among those aged 6–11 years^6^^,^^39^^,^^42^ for females than for males. In addition, a prior study of those aged 12–18 years found that females were more likely to receive BP-lowering medications than males among those with secondary hypertension after adjusting for obesity.^43^ For guideline-recommended medications,^13^ higher percentages of prescription fills among males than among females were observed for all age groups, which is consistent with findings of higher prevalence of increased BP among males than among females.

The notable increase in prescription fills among adolescent females (aged 13–17 years) was primarily driven by 4 medications: spironolactone, clonidine, propranolol, and prazosin (Appendix Tables 4 and 5, available online). These 4 BP-lowering medications were included in the Fourth Report^15^^,^^44^ for outpatient management of pediatric hypertension but were excluded from the 2017 AAP guideline for the outpatient management of chronic hypertension. Although these 4 medications were not included in the 2017 AAP guideline, they may be prescribed for hypertension or other conditions. For example, the 2017 AAP guideline indicates that it would be reasonable to use aldosterone receptor antagonists (such as spironolactone) to treat resistant hypertension among children in a similar manner as in adults,^45^ and existing literature highlights the frequent combination of spironolactone with oral contraceptive pills to treat adolescent PCOS.^27^ Clonidine is approved by the FDA for the treatment of hypertension,^46^ but it may also be prescribed to treat insomnia among children and adolescents because there are no FDA-approved sleep medications for pediatric individuals.^47^^,^^48^ Although propranolol is an effective treatment for reducing BP,^26^ it has also been recommended as a pharmacologic alternative for conditions such as migraines,^15^^,^^44^ anxiety,^49^ or ASD.^26^ Prazosin, a peripheral antagonist, is also prescribed for pediatric post-traumatic stress disorder–associated nightmares and sleep disturbances.^50^^,^^51^

Recent prevalence estimates and physiologic differences between adolescent males and females partly explain the rise in prescription fills for these 4 BP-lowering medications among adolescent females. For example, a population-based U.S. study has highlighted an upward trend of prevalence and incidence of PCOS among U.S. adolescents aged 16–20 years in recent years that may have contributed to the increase in prescription fills for spironolactone.^52^ Prior studies reveal no sex difference in the incidence of insomnia up to the median age of 11 years; however, after puberty, there is an uptick in the incidence of insomnia among adolescent females.^53^^,^^54^ Among high-school students in the U.S., the prevalence of short sleep duration has increased, and it is higher among female students than among male students.^55^^,^^56^ An increasing prevalence of hypertension; obesity; anxiety^49^; and other conditions such as PCOS, insomnia, short sleep duration, post-traumatic stress disorder, or ASD may have contributed to the increases in BP-lowering prescription fills among females aged 13–17 years.

In contrast to increasing trends observed for the 4 medications named earlier and for BP-lowering prescription fills overall, this study documented a declining trend for prescription fills for most of the guideline-recommended medications.^13^ Although these estimates may underreport guideline-recommended medications, because combination drugs were not included, only 2 guideline-recommended medications—enalapril and losartan—were among BP-lowering medications with substantial increases in prescription fills during 2017–2023 (Appendix Table 5, available online). Increases in these 2 medications could reflect prescriptions for pediatric hypertension; a recent network meta-analysis found enalapril, lisinopril, and losartan superior to placebo and other medications, such as eplerenone, in reducing BP measures among children and adolescents.^57^ However, the small fraction of BP-lowering prescription fills for guideline-recommended medications as well as the decline in fills for guideline-recommended medications in the context of the overall increase in prescription fills for BP-lowering medications might indicate treatment of health conditions other than pediatric hypertension. One such example could be anxiety, because there was a significant increase in the prevalence of anxiety problems among U.S. children aged 3–17 years from 2016 to 2020.^49^ Decline in guideline-recommended medications could also be due to the lack of guideline awareness and underdiagnosis of pediatric hypertension.^58^ This study reveals the complexity surrounding the prescription of BP-lowering medications and pharmacologic treatment of pediatric hypertension, which are exacerbated by the lack of long-term outcome data and paucity of pediatric-specific clinical trials.^17^^,^^59^

### Limitations

This study has several limitations. First, analysis provided trends of prescription fills for outpatient retail orders only, excluding mail orders or other prescription sources. This may have led to an underestimation of the total number of BP-lowering prescriptions filled. However, prior work has found that 96.4% of prescription fills for U.S. children were from retail pharmacies in 2019, whereas only 1.3% of total prescriptions were mail-order prescriptions.^60^ Second, this study could not account for dosage or frequency of use. For example, some BP-lowering medications may have only been filled once by a given patient during a measurement year, and it could not be ascertained whether or how the medications were taken. Third, data for important factors, such as income, race, and region, that may influence prescription patterns were not available. Fourth, this study could not identify the underlying conditions or diagnoses for which these medications were prescribed, and many of these medications could have been filled for purposes other than elevated BP or hypertension.^44^ Future studies may aim to link prescription data with clinical data to better understand the reasons behind the observed trends.

## CONCLUSIONS

This study provided the first national-level estimates of pediatric BP-lowering prescription fills in the U.S. using the most recent outpatient prescription database. The study complements existing literature on pediatric hypertension by reporting patterns of prescription fills by sex and age groups that helped identify populations with higher utilization of BP-lowering medications over time. This includes the first-ever nationwide estimate of BP-lowering medications among children aged 3–7 years, who are not assessed in other national datasets. There was an increasing trend of BP-lowering prescription fills among U.S. children and adolescents aged 3–17 years during 2017–2023, particularly among females aged 13–17 years. Understanding the landscape of pediatric BP-lowering medication prescribing practices is helpful for both clinicians and public health practitioners. BP-lowering prescription fills may indicate pharmacologic treatment of pediatric hypertension, related comorbidities, or other conditions unrelated to BP. The findings of this study might inform therapeutic decision making and encourage clinicians to initiate indicated pharmacotherapy in pediatric populations, could impact the development of future management guidelines, and could foster better-informed practices for pediatric hypertension.

## Appendix Supplementary materials

Image, application 1

## Footnotes

Supplementary material associated with this article can be found in the online version at doi:10.1016/j.focus.2025.100356.
