# Supplementary material for: Trends in Pediatric Blood Pressure–Lowering Prescription Fills During 2017–2023
Source: AJPM Focus. 2025 Apr 22;4(4):100356. doi: 10.1016/j.focus.2025.100356 (PMC12240082; doi:10.1016/j.focus.2025.100356)
Supplement: Supplementary file 1 [file mmc1.docx]

**LIST OF TABLES IN ONLINE APPEDIX**

Appendix Table1: List of 113 BP-lowering medications from 21 drug classes, IQVIA Total Patient Tracker, 2017–2023

Appendix Table2: Annual total number and population percentage of unique individuals aged 3 to 17 years with prescription fills for generic BP-lowering medications during 2017–2023 overall and by sex and age groups, IQVIA Total Patient Tracker, 2017–2023

Appendix Table3: Annual total number and population percentage of unique individuals aged 3 to 17 years with prescription fills for generic BP-lowering medications mentioned in 2017 AAP guideline for outpatient management of chronic hypertension during 2017–2023 overall and by sex and age groups, IQVIA Total Patient Tracker, 2017–2023

Appendix Table4: Annual total number of unique individuals aged 3 to 17 years with prescription fills for generic BP-lowering medications for each of 113 products from 21 drug classes, IQVIA Total Patient Tracker, 2017–2023

Appendix Table5: Annual total number of unique individuals aged 3 to 17 years with prescription fills for generic BP-lowering medications that had greatest increase (>500) in number of individuals filling prescriptions during 2017–2023, IQVIA Total Patient Tracker, 2017–2023

**ONLINE-ONLY APPEDIX**

**Appendix Table1:** List of 113 BP-lowering medications from 21 drug classes, IQVIA Total Patient Tracker, 2017–2023

| **Serial No.** | **Drug class** | **Medication** | **2017 AAP guideline recommended** |
| --- | --- | --- | --- |
| 1 | ACE INHIBITOR, ALONE/OTHER | BENAZEPRIL HCL | Yes |
| 2 |  | CAPTOPRIL | Yes |
| 3 |  | ENALAPRIL MAL | Yes |
| 4 |  | FOSINOPRIL SOD | Yes |
| 5 |  | LISINOPRIL | Yes |
| 6 |  | MOEXIPRIL HCL | No |
| 7 |  | PERINDOPRIL ERBUMINE | No |
| 8 |  | QUINAPRIL HCL | Yes |
| 9 |  | RAMIPRIL | Yes |
| 10 |  | TRANDOLAPRIL | No |
| 11 |  | AMLODIP BES/BENAZ HCL | No |
| 12 |  | TRANDOL/VERAP HCL | No |
| 13 | ACE INHIBITOR, WITH DIURETICS | BENAZEPRIL/HCTZ | No |
| 14 |  | CAPTOPRIL/HCTZ | No |
| 15 |  | ENALAPRIL MAL/HCTZ | No |
| 16 |  | FOSINOPRIL/HCTZ | No |
| 17 |  | LISINOPRIL/HCTZ | No |
| 18 |  | MOEXIPRIL HCL/HCTZ | No |
| 19 |  | QUINAPRIL HCL/HCTZ | No |
| 20 | ALPHA BLOCKERS,ALONE,COMB | DOXAZOSIN MESY | No |
| 21 |  | PHENOXYBENZAMINE | No |
| 22 |  | PRAZOSIN HCL | No |
| 23 |  | TERAZOSIN HCL | No |
| e24 | ALPHA-BETA BLOCKER | CARVEDILOL | No |
| 25 |  | CARVEDILOL PHOSPH | No |
| 26 |  | LABETALOL HCL | No |
| 27 | ANGIO II ANTAG,ALONE | CANDESARTAN CIL | Yes |
| 28 |  | EPROSARTAN MESY | No |
| 29 |  | IRBESARTAN | Yes |
| 30 |  | LOSARTAN POT | Yes |
| 31 |  | OLMESARTAN MEDOX | Yes |
| 32 |  | TELMISARTAN | No |
| 33 |  | VALSARTAN | Yes |
| 34 | ANGIO II ANTAG,WITH CCB | AMLODIP BES/OLMESAR | No |
| 35 |  | AMLODIP BES/VALSAR | No |
| 36 |  | TELMISARTAN/AMLODIP | No |
| 37 | ANGIO II ANTAG,WITH/CCB/DIURETICS | AMLODIP/VALS/HCTZ | No |
| 38 |  | OLMSRTN/AMLDPN/HCTZ | No |
| 39 | ANGIO II ANTAG,WITH DIURETICS | CANDESARTAN-HCTZ | No |
| 40 |  | IRBESARTAN/HCTZ | No |
| 41 |  | LOSARTAN POT/HCTZ | No |
| 42 |  | OLMESARTAN/HCTZ | No |
| 43 |  | TELMISARTAN/HCTZ | No |
| 44 |  | VALSARTAN-HCTZ | No |
| 45 | BETA BLOCKERS  BETA BLOCKERS | ACEBUTOLOL HCL | No |
| 46 |  | ATENOLOL | No |
| 47 |  | BETAXOLOL HCL | No |
| 48 |  | BISOPROLOL FUM | No |
| 49 |  | BISOPROLOL FUMARATE | No |
| 50 |  | BREVIBLOC | No |
| 51 |  | METOPROLOL SUCCIN | No |
| 52 |  | METOPROLOL TART | No |
| 53 |  | NADOLOL | No |
| 54 |  | NEBIVOLOL HCL | No |
| 55 |  | PINDOLOL | No |
| 56 |  | PROPRANOLOL HCL | No |
| 57 |  | TIMOLOL MAL | No |
| 58 | BETA/ ALPHA-BETA BLOCKER WITH DIURETICS | ATENOLOL/CHLORTHAL | No |
| 59 |  | BISOPROLOL FUM/HCTZ | No |
| 60 |  | METOPROLOL/HCTZ | No |
| 61 |  | METOPROLOL/HCTZ ER | No |
| 62 |  | NADOLOL/BENDROFLUM | No |
| 63 |  | PROPRANOLOL/HCTZ | No |
| 64 | CALCIUM BLOCKERS | AMLODIPINE BESY | Yes |
| 65 |  | DILTIAZEM 24HR | No |
| 66 |  | DILTIAZEM HCL | No |
| 67 |  | DILTIAZEM SR | No |
| 68 |  | DILTIAZEM XR | No |
| 69 |  | FELODIPINE ER | Yes |
| 70 |  | ISRADIPINE | Yes |
| 71 |  | LEVAMLODIPINE MALEATE | No |
| 72 |  | MATZIM LA | No |
| 73 |  | NICARDIPINE HCL | No |
| 74 |  | NIFEDIPINE | No |
| 75 |  | NIFEDIPINE ER | Yes |
| 76 |  | NIMODIPINE | No |
| 77 |  | NISOLDIPINE | No |
| 78 |  | VERAPAMIL HCL | No |
| 79 |  | VERAPAMIL SR | No |
| 80 |  | VERAPAMIL SR PM | No |
| 81 | CENTRAL ACT AGT,ALONE,COMB | CLONIDINE | No |
| 82 |  | CLONIDINE HCL | No |
| 83 |  | CLONIDINE/CHLORTHL | No |
| 84 |  | GUANFACINE HCL | No |
| 85 |  | METHYLDOPA | No |
| 86 |  | METHYLDOPA/HCTZ | No |
| 87 | DIRECT RENIN INHIB,ALONE | ALISKIREN HEMIFUM | No |
| 88 | DIURETICS,COMB | AMILORIDE HCL/HCTZ | No |
| 89 |  | MAXZIDE 25 | No |
| 90 |  | SPIRONOLACTONE/HCT | No |
| 91 |  | TRIAMTERENE/HCTZ | No |
| 92 | DIURETICS,LOOP | BUMETANIDE | No |
| 93 |  | ETHACRYNATE SOD | No |
| 94 |  | ETHACRYNIC ACID | No |
| 95 |  | FUROSEMIDE | No |
| 96 |  | TORSEMIDE | No |
| 97 | DIURETICS,POT SPARING | AMILORIDE HCL | No |
| 98 |  | SPIRONOLACTONE | No |
| 99 |  | TRIAMTERENE | No |
| 100 | DIURETICS,THIAZIDE & RELATED | CHLOROTHIAZIDE | Yes |
| 101 |  | CHLOROTHIAZIDE SOD | Yes |
| 102 |  | CHLORTHALIDONE | Yes |
| 103 |  | HYDROCHLOROTHIAZIDE | Yes |
| 104 |  | INDAPAMIDE | No |
| 105 |  | METHYCLOTHIAZIDE | No |
| 106 |  | METOLAZONE | No |
| 107 | SEL ALDOSTERONE RECPT ANTAG | EPLERENONE | No |
| 108 | VASC/ANTIHYPERLIPIDEMIC COMB | AMLODIP BES/ATORVAST | No |
| 109 | VASCULAR AGENTS, OTHER | HYDRALAZINE HCL | No |
| 110 |  | METYROSINE | No |
| 111 |  | MINOXIDIL | No |
| 112 |  | PHENTOLAMINE MESY | No |
| 113 |  | RESERPINE | No |

Abbreviations: ; ACE, angiotensin-converting enzyme; ACT, acting; AGT, agent; ANTAG, antagonist; BES(Y), besylate; CCB, calcium channel blocker; CIL, cilexitil; COMB, combination; Diff, difference; ER, extended release; FUM, fumarate; HCL, hydrochloride; HCTZ, hydrochlorothiazide; LA, long-acting; MAL, maleate; MEDOX, medoxomil; MESY, mesylate; OLMESAR, olmesartan; POT, potassium; PM, post meridem; RECPT, receptor; SEL, selective; SOD, sodium; SR, sustained release; VALSAR, valsartan; VASC, vascular; XR, extended release

**Appendix Table2:** Annual total number and population percentage of unique individuals aged 3 to 17 years with prescription fills for generic BP-lowering medications during 2017–2023 overall and by sex and age groups, IQVIA Total Patient Tracker, 2017–2023^a^

| Group | Year | Count | US population | Population percent | 95% lower CI | 95%  higher CI | Change (%) |
| --- | --- | --- | --- | --- | --- | --- | --- |
| ***Overall samples for all ages (3–17 years)^b^*** | | | | | | | |
| Total | 2017 | 1,190,800 | 61719056 | 1.93% | 1.88% | 1.98% | Reference |
| Total | 2018 | 1,196,266 | 61646797 | 1.94% | 1.89% | 1.99% | 0.58% |
| Total | 2019 | 1,191,667 | 61504455 | 1.94% | 1.89% | 1.99% | 0.42% |
| Total | 2020 | 1,181,355 | 61460194 | 1.92% | 1.87% | 1.97% | -0.38% |
| Total | 2021 | 1,244,673 | 62531576 | 1.99% | 1.94% | 2.04% | 3.17% |
| Total | 2022 | 1,280,570 | 61485535 | 2.08% | 2.03% | 2.13% | 7.95% |
| Total | 2023 | 1,284,456 | 61485535 | 2.09% | 2.04% | 2.14% | 8.27% |
| Male | 2017 | 749,932 | 31518277 | 2.38% | 2.31% | 2.45% | Reference |
| Male | 2018 | 741,437 | 31480886 | 2.36% | 2.28% | 2.43% | -1.02% |
| Male | 2019 | 729,566 | 31409422 | 2.32% | 2.25% | 2.39% | -2.38% |
| Male | 2020 | 704,328 | 31391063 | 2.24% | 2.17% | 2.31% | -5.70% |
| Male | 2021 | 715,512 | 31997800 | 2.24% | 2.17% | 2.31% | -6.02% |
| Male | 2022 | 733,017 | 31468659 | 2.33% | 2.26% | 2.40% | -2.10% |
| Male | 2023 | 730,257 | 31468659 | 2.32% | 2.26% | 2.38% | -2.47% |
| Female | 2017 | 449,489 | 30200779 | 1.49% | 1.44% | 1.54% | Reference |
| Female | 2018 | 456,162 | 30165911 | 1.51% | 1.47% | 1.56% | 1.60% |
| Female | 2019 | 462,009 | 30095033 | 1.54% | 1.49% | 1.58% | 3.15% |
| Female | 2020 | 475,883 | 30069131 | 1.58% | 1.53% | 1.63% | 6.34% |
| Female | 2021 | 527,219 | 30533776 | 1.73% | 1.67% | 1.78% | 16.01% |
| Female | 2022 | 546,337 | 30016876 | 1.82% | 1.76% | 1.88% | 22.29% |
| Female | 2023 | 553,516 | 30016876 | 1.84% | 1.79% | 1.90% | 23.90% |
| ***Stratified samples by sex and age groups*** | | | | | | | |
| **Males** |  |  |  |  |  |  |  |
| 3 to 7 | 2017 | 153123 | 10269729 | 1.49% | 1.42% | 1.56% | Reference |
| 3 to 7 | 2018 | 151279 | 10300135 | 1.47% | 1.40% | 1.53% | -1.50% |
| 3 to 7 | 2019 | 147306 | 10279852 | 1.43% | 1.37% | 1.50% | -3.89% |
| 3 to 7 | 2020 | 142674 | 10256664 | 1.39% | 1.33% | 1.45% | -6.70% |
| 3 to 7 | 2021 | 142457 | 10213921 | 1.39% | 1.33% | 1.46% | -6.46% |
| 3 to 7 | 2022 | 147613 | 9983676 | 1.48% | 1.41% | 1.54% | -0.84% |
| 3 to 7 | 2023 | 154670 | 9983676 | 1.55% | 1.48% | 1.62% | 3.90% |
| 8 to 12 | 2017 | 343202 | 10571648 | 3.25% | 3.13% | 3.36% | Reference |
| 8 to 12 | 2018 | 334098 | 10549398 | 3.17% | 3.05% | 3.28% | -2.45% |
| 8 to 12 | 2019 | 329201 | 10512505 | 3.13% | 3.02% | 3.24% | -3.54% |
| 8 to 12 | 2020 | 314599 | 10448829 | 3.01% | 2.90% | 3.12% | -7.26% |
| 8 to 12 | 2021 | 311709 | 10589541 | 2.94% | 2.84% | 3.05% | -9.33% |
| 8 to 12 | 2022 | 316309 | 10392126 | 3.04% | 2.93% | 3.15% | -6.24% |
| 8 to 12 | 2023 | 311676 | 10392126 | 3.00% | 2.89% | 3.11% | -7.62% |
| 13 to 17 | 2017 | 276212 | 10676900 | 2.59% | 2.49% | 2.68% | Reference |
| 13 to 17 | 2018 | 278154 | 10631353 | 2.62% | 2.52% | 2.71% | 1.13% |
| 13 to 17 | 2019 | 275161 | 10617065 | 2.59% | 2.50% | 2.69% | 0.18% |
| 13 to 17 | 2020 | 269429 | 10685570 | 2.52% | 2.43% | 2.61% | -2.53% |
| 13 to 17 | 2021 | 282965 | 11194338 | 2.53% | 2.44% | 2.62% | -2.29% |
| 13 to 17 | 2022 | 290809 | 11092857 | 2.62% | 2.53% | 2.72% | 1.34% |
| 13 to 17 | 2023 | 286716 | 11092857 | 2.58% | 2.49% | 2.68% | -0.09% |
| **Females** |  |  |  |  |  |  |  |
| 3 to 7 | 2017 | 69268 | 9822038 | 0.71% | 0.67% | 0.74% | Reference |
| 3 to 7 | 2018 | 66364 | 9842335 | 0.67% | 0.64% | 0.71% | -4.39% |
| 3 to 7 | 2019 | 63601 | 9829963 | 0.65% | 0.61% | 0.68% | -8.26% |
| 3 to 7 | 2020 | 64713 | 9806967 | 0.66% | 0.62% | 0.70% | -6.43% |
| 3 to 7 | 2021 | 65600 | 9759938 | 0.67% | 0.64% | 0.71% | -4.69% |
| 3 to 7 | 2022 | 66750 | 9549310 | 0.70% | 0.66% | 0.74% | -0.88% |
| 3 to 7 | 2023 | 69006 | 9549310 | 0.72% | 0.68% | 0.76% | 2.47% |
| 8 to 12 | 2017 | 158212 | 10139195 | 1.56% | 1.49% | 1.63% | Reference |
| 8 to 12 | 2018 | 154409 | 10117073 | 1.53% | 1.46% | 1.59% | -2.19% |
| 8 to 12 | 2019 | 153158 | 10074151 | 1.52% | 1.45% | 1.59% | -2.57% |
| 8 to 12 | 2020 | 153313 | 10012768 | 1.53% | 1.46% | 1.60% | -1.87% |
| 8 to 12 | 2021 | 157961 | 10092839 | 1.57% | 1.50% | 1.63% | 0.30% |
| 8 to 12 | 2022 | 159573 | 9903066 | 1.61% | 1.54% | 1.68% | 3.27% |
| 8 to 12 | 2023 | 159870 | 9903066 | 1.61% | 1.54% | 1.69% | 3.46% |
| 13 to 17 | 2017 | 231156 | 10239546 | 2.26% | 2.17% | 2.35% | Reference |
| 13 to 17 | 2018 | 244229 | 10206503 | 2.39% | 2.30% | 2.49% | 6.00% |
| 13 to 17 | 2019 | 254331 | 10190919 | 2.50% | 2.41% | 2.59% | 10.55% |
| 13 to 17 | 2020 | 266853 | 10249396 | 2.60% | 2.51% | 2.70% | 15.33% |
| 13 to 17 | 2021 | 312619 | 10680999 | 2.93% | 2.82% | 3.03% | 29.65% |
| 13 to 17 | 2022 | 329820 | 10564500 | 3.12% | 3.01% | 3.23% | 38.29% |
| 13 to 17 | 2023 | 334579 | 10564500 | 3.17% | 3.05% | 3.28% | 40.29% |

Abbreviations: CI, confidence interval

^a^ This table presents the trends of prescription fills for BP-lowering generic medications among children and adolescents aged 3 to 17 years from IQVIA’s Total Patient Tracker database. Drug prescriptions for unspecified sex and age groups were excluded.

^b^ The aggregate count of individuals with BP-lowering prescription fills for sex and age groups are generated separately for age groups 3 to 17, excluding results for unspecified sex and age groups. The total aggregate numbers are lower than the sum of numbers from subgroups as the deduplication process removes multiple entries for the same person appearing in different age bands while removing all conflicting/missing reports of sex and age groups.

**Appendix Table3:** Annual total number and population percentage of unique individuals aged 3 to 17 years with prescription fills for generic BP-lowering medications mentioned in 2017 AAP guideline for outpatient management of chronic hypertension during 2017–2023 overall and by sex and age groups, IQVIA Total Patient Tracker, 2017–2023^a^

| Group | Year | Count | Population | Population percent | 95%  lower CI | 95%  higher CI | Change (%) |
| --- | --- | --- | --- | --- | --- | --- | --- |
| ***Overall samples for all ages (3–17 years)^b^*** | | | | | |  |  |
| Total | 2017 | 120838 | 61719056 | 0.20% | 0.19% | 0.21% | Reference |
| Total | 2018 | 117050 | 61646797 | 0.19% | 0.18% | 0.20% | -3.02% |
| Total | 2019 | 100888 | 61504455 | 0.16% | 0.16% | 0.17% | -16.22% |
| Total | 2020 | 96565 | 61460194 | 0.16% | 0.15% | 0.17% | -19.75% |
| Total | 2021 | 102322 | 62531576 | 0.16% | 0.16% | 0.17% | -16.42% |
| Total | 2022 | 103875 | 61485535 | 0.17% | 0.16% | 0.18% | -13.71% |
| Total | 2023 | 100948 | 61485535 | 0.16% | 0.16% | 0.17% | -16.14% |
| Male | 2017 | 70743 | 31518277 | 0.22% | 0.21% | 0.24% | Reference |
| Male | 2018 | 67866 | 31480886 | 0.22% | 0.20% | 0.23% | -3.95% |
| Male | 2019 | 58724 | 31409422 | 0.19% | 0.18% | 0.20% | -16.70% |
| Male | 2020 | 56307 | 31391063 | 0.18% | 0.17% | 0.19% | -20.08% |
| Male | 2021 | 60079 | 31997800 | 0.19% | 0.18% | 0.20% | -16.35% |
| Male | 2022 | 61023 | 31468659 | 0.19% | 0.18% | 0.21% | -13.60% |
| Male | 2023 | 59226 | 31468659 | 0.19% | 0.18% | 0.20% | -16.15% |
| Female | 2017 | 51861 | 30200779 | 0.17% | 0.16% | 0.18% | Reference |
| Female | 2018 | 48880 | 30165911 | 0.16% | 0.15% | 0.17% | -5.64% |
| Female | 2019 | 41575 | 30095033 | 0.14% | 0.13% | 0.15% | -19.55% |
| Female | 2020 | 39595 | 30069131 | 0.13% | 0.12% | 0.14% | -23.32% |
| Female | 2021 | 41549 | 30533776 | 0.14% | 0.13% | 0.15% | -20.76% |
| Female | 2022 | 42263 | 30016876 | 0.14% | 0.13% | 0.15% | -18.01% |
| Female | 2023 | 41119 | 30016876 | 0.14% | 0.13% | 0.15% | -20.23% |
| ***Stratified samples by sex and age groups*** | | | |  |  |  |  |
| **Males** |  |  |  |  |  |  |  |
| 3 to 7 | 2017 | 7386 | 10064047 | 0.07% | 0.06% | 0.08% | Reference |
| 3 to 7 | 2018 | 6402 | 10064047 | 0.06% | 0.06% | 0.07% | -13.58% |
| 3 to 7 | 2019 | 4678 | 10064047 | 0.05% | 0.04% | 0.05% | -36.72% |
| 3 to 7 | 2020 | 4465 | 10064047 | 0.04% | 0.04% | 0.05% | -39.47% |
| 3 to 7 | 2021 | 5369 | 10064047 | 0.05% | 0.05% | 0.06% | -26.92% |
| 3 to 7 | 2022 | 6460 | 10064047 | 0.06% | 0.06% | 0.07% | -10.03% |
| 3 to 7 | 2023 | 6527 | 10064047 | 0.07% | 0.06% | 0.07% | -9.10% |
| 8 to 12 | 2017 | 19296 | 10782507 | 0.18% | 0.17% | 0.20% | Reference |
| 8 to 12 | 2018 | 18526 | 10782507 | 0.18% | 0.16% | 0.19% | -3.79% |
| 8 to 12 | 2019 | 16412 | 10782507 | 0.16% | 0.14% | 0.17% | -14.46% |
| 8 to 12 | 2020 | 15682 | 10782507 | 0.15% | 0.14% | 0.16% | -17.77% |
| 8 to 12 | 2021 | 15852 | 10782507 | 0.15% | 0.14% | 0.16% | -17.98% |
| 8 to 12 | 2022 | 15996 | 10782507 | 0.15% | 0.14% | 0.17% | -15.67% |
| 8 to 12 | 2023 | 15557 | 10782507 | 0.15% | 0.14% | 0.16% | -17.98% |
| 13 to 17 | 2017 | 43749 | 11082391 | 0.41% | 0.38% | 0.44% | Reference |
| 13 to 17 | 2018 | 42894 | 11082391 | 0.40% | 0.38% | 0.43% | -1.53% |
| 13 to 17 | 2019 | 38286 | 11082391 | 0.36% | 0.34% | 0.38% | -11.99% |
| 13 to 17 | 2020 | 36827 | 11082391 | 0.34% | 0.32% | 0.37% | -15.89% |
| 13 to 17 | 2021 | 39552 | 11082391 | 0.35% | 0.33% | 0.38% | -13.77% |
| 13 to 17 | 2022 | 39360 | 11082391 | 0.35% | 0.33% | 0.38% | -13.40% |
| 13 to 17 | 2023 | 38136 | 11082391 | 0.34% | 0.32% | 0.37% | -16.10% |
| **Females** |  |  |  |  |  |  |  |
| 3 to 7 | 2017 | 5799 | 9646634 | 0.06% | 0.05% | 0.07% | Reference |
| 3 to 7 | 2018 | 4681 | 9646634 | 0.05% | 0.04% | 0.05% | -19.44% |
| 3 to 7 | 2019 | 3436 | 9646634 | 0.03% | 0.03% | 0.04% | -40.80% |
| 3 to 7 | 2020 | 3316 | 9646634 | 0.03% | 0.03% | 0.04% | -42.74% |
| 3 to 7 | 2021 | 4004 | 9646634 | 0.04% | 0.04% | 0.05% | -30.51% |
| 3 to 7 | 2022 | 5058 | 9646634 | 0.05% | 0.05% | 0.06% | -10.30% |
| 3 to 7 | 2023 | 4944 | 9646634 | 0.05% | 0.05% | 0.06% | -12.32% |
| 8 to 12 | 2017 | 14704 | 10293477 | 0.15% | 0.13% | 0.16% | Reference |
| 8 to 12 | 2018 | 14259 | 10293477 | 0.14% | 0.13% | 0.15% | -2.81% |
| 8 to 12 | 2019 | 12513 | 10293477 | 0.12% | 0.11% | 0.14% | -14.35% |
| 8 to 12 | 2020 | 11702 | 10293477 | 0.12% | 0.11% | 0.13% | -19.41% |
| 8 to 12 | 2021 | 11636 | 10293477 | 0.12% | 0.10% | 0.13% | -20.50% |
| 8 to 12 | 2022 | 11934 | 10293477 | 0.12% | 0.11% | 0.13% | -16.90% |
| 8 to 12 | 2023 | 11367 | 10293477 | 0.11% | 0.10% | 0.13% | -20.85% |
| 13 to 17 | 2017 | 31036 | 10551612 | 0.30% | 0.28% | 0.33% | Reference |
| 13 to 17 | 2018 | 30007 | 10551612 | 0.29% | 0.27% | 0.32% | -3.00% |
| 13 to 17 | 2019 | 26139 | 10551612 | 0.26% | 0.24% | 0.28% | -15.38% |
| 13 to 17 | 2020 | 25038 | 10551612 | 0.24% | 0.23% | 0.26% | -19.40% |
| 13 to 17 | 2021 | 26332 | 10551612 | 0.25% | 0.23% | 0.27% | -18.66% |
| 13 to 17 | 2022 | 25925 | 10551612 | 0.25% | 0.23% | 0.26% | -19.04% |
| 13 to 17 | 2023 | 25474 | 10551612 | 0.24% | 0.22% | 0.26% | -20.44% |

Abbreviations: CI, confidence interval

^a^ This table presents the trend of prescription fills for BP-lowering generic medications among children and adolescents aged 3 to 17 years from IQVIA’s Total Patient Tracker database for medications mentioned in Table 17 of the 2017 AAP guideline. Drug prescriptions for unspecified sex and age groups were excluded.

^b^ The aggregate count of sex and age groups are generated separately for age groups 3 to 17, excluding results for unspecified sex and age groups. The total aggregate numbers are lower than the sum of numbers from subgroups as the deduplication process removes multiple entries for the same person appearing in different age bands while removing all conflicting/missing reports of sex and age groups.

**Appendix Table4:** Annual total number of unique individuals aged 3 to 17 years with prescription fills for generic BP-lowering medications for each of 113 products from 21 drug classes, IQVIA Total Patient Tracker, 2017–2023^a^

| **Drug class** | **Product brand** | **Age group** | **2017** | **2018** | **2019** | **2020** | **2021** | **2022** | **2023** | **Diff^b^** |
| --- | --- | --- | --- | --- | --- | --- | --- | --- | --- | --- |
| ACE INHIBITOR, ALONE | BENAZEPRIL HCL | 3 - 7 | 1,603 | 1,351 | 983 | 989 | 899 | 839 | 767 | -836 |
|  | BENAZEPRIL HCL | 8 - 12 | 3,245 | 3,684 | 3,214 | 3,230 | 2,852 | 2,656 | 2,126 | -1119 |
|  | BENAZEPRIL HCL | 13 - 17 | 3,848 | 4,006 | 2,132 | 2,240 | 2,446 | 2,015 | 2,047 | -1801 |
|  | CAPTOPRIL | 3 - 7 | 122 | 104 | 88 | 77 | 52 | 49 | 52 | -69 |
|  | CAPTOPRIL | 8 - 12 | 93 | 83 | 75 | 54 | 57 | 55 | 49 | -43 |
|  | CAPTOPRIL | 13 - 17 | 189 | 133 | 89 | 68 | 54 | 58 | 51 | -138 |
|  | ENALAPRIL MAL | 3 - 7 | 4,866 | 3,896 | 3,128 | 2,866 | 5,004 | 6,800 | 6,694 | 1828 |
|  | ENALAPRIL MAL | 8 - 12 | 11,332 | 11,124 | 9,734 | 9,017 | 8,901 | 8,963 | 8,415 | -2916 |
|  | ENALAPRIL MAL | 13 - 17 | 14,444 | 13,461 | 10,371 | 9,998 | 9,668 | 9,132 | 8,377 | -6067 |
|  | FOSINOPRIL SOD | 3 - 7 | 12 | 15 | 5 | 8 |  |  |  |  |
|  | FOSINOPRIL SOD | 8 - 12 | 5 | 3 | 2 |  | 2 |  | 1 | -4 |
|  | FOSINOPRIL SOD | 13 - 17 | 34 | 39 | 13 | 11 | 17 | 9 | 9 | -26 |
|  | LISINOPRIL | 3 - 7 | 2,664 | 2,473 | 1,759 | 1,654 | 1,502 | 1,436 | 1,325 | -1339 |
|  | LISINOPRIL | 8 - 12 | 10,232 | 9,780 | 8,776 | 8,387 | 8,419 | 8,344 | 7,914 | -2318 |
|  | LISINOPRIL | 13 - 17 | 32,140 | 31,843 | 30,538 | 29,132 | 30,855 | 30,224 | 28,252 | -3888 |
|  | MOEXIPRIL HCL | 3 - 7 | 1 | 1 |  |  |  |  |  |  |
|  | MOEXIPRIL HCL | 8 - 12 | 0 |  |  |  |  |  |  |  |
|  | MOEXIPRIL HCL | 13 - 17 | 1 | 1 |  |  |  |  |  |  |
|  | PERINDOPRIL ERBUMINE | 3 - 7 |  | 1 | 4 | 1 | 2 |  |  |  |
|  | PERINDOPRIL ERBUMINE | 8 - 12 | 6 |  |  | 1 | 5 | 4 | 2 | -4 |
|  | PERINDOPRIL ERBUMINE | 13 - 17 | 4 | 6 | 4 | 5 | 5 | 6 | 5 | 1 |
|  | QUINAPRIL HCL | 3 - 7 | 5 | 1 | 0 | 1 | 1 | 1 | 2 | -3 |
|  | QUINAPRIL HCL | 8 - 12 | 12 | 11 | 9 | 4 | 6 | 5 | 8 | -4 |
|  | QUINAPRIL HCL | 13 - 17 | 60 | 41 | 35 | 41 | 22 | 28 | 9 | -51 |
|  | RAMIPRIL | 3 - 7 | 19 | 10 | 8 | 6 | 4 | 7 | 1 | -19 |
|  | RAMIPRIL | 8 - 12 | 40 | 41 | 28 | 29 | 21 | 16 | 28 | -12 |
|  | RAMIPRIL | 13 - 17 | 231 | 189 | 126 | 110 | 96 | 74 | 67 | -165 |
|  | TRANDOLAPRIL | 3 - 7 | 2 | 1 | 0 | 0 |  |  |  |  |
|  | TRANDOLAPRIL | 8 - 12 | 0 | 0 |  | 1 |  | 0 |  |  |
|  | TRANDOLAPRIL | 13 - 17 | 2 | 1 |  |  |  | 3 |  |  |
| ACE INHIBITOR,OTHER | AMLODIP BES/BENAZ HCL | 3 - 7 | 9 | 8 | 11 | 11 | 3 | 4 | 6 | -3 |
|  | AMLODIP BES/BENAZ HCL | 8 - 12 | 69 | 56 | 45 | 30 | 30 | 52 | 46 | -23 |
|  | AMLODIP BES/BENAZ HCL | 13 - 17 | 321 | 318 | 264 | 259 | 246 | 242 | 226 | -95 |
| ACE INHIBITOR, WITH DIURETIC | BENAZEPRIL/HCTZ | 3 - 7 | 1 | 7 | 6 | 5 | 2 | 1 |  |  |
|  | BENAZEPRIL/HCTZ | 8 - 12 | 1 | 2 | 2 | 4 | 16 | 0 | 3 | 3 |
|  | BENAZEPRIL/HCTZ | 13 - 17 | 58 | 48 | 37 | 21 | 16 | 19 | 23 | -34 |
|  | CAPTOPRIL/HCTZ | 13 - 17 |  | 2 |  |  |  |  |  |  |
|  | ENALAPRIL MAL/HCTZ | 3 - 7 | 3 | 7 | 2 | 3 | 0 |  | 2 | -1 |
|  | ENALAPRIL MAL/HCTZ | 8 - 12 | 24 | 20 | 6 | 1 | 3 | 1 | 6 | -19 |
|  | ENALAPRIL MAL/HCTZ | 13 - 17 | 77 | 73 | 61 | 36 | 40 | 37 | 19 | -59 |
|  | FOSINOPRIL/HCTZ | 3 - 7 |  | 0 |  |  |  |  |  |  |
|  | FOSINOPRIL/HCTZ | 13 - 17 | 1 | 2 |  |  |  |  |  |  |
|  | LISINOPRIL/HCTZ | 3 - 7 | 15 | 21 | 11 | 20 | 20 | 7 | 5 | -10 |
|  | LISINOPRIL/HCTZ | 8 - 12 | 320 | 307 | 181 | 149 | 123 | 126 | 110 | -210 |
|  | LISINOPRIL/HCTZ | 13 - 17 | 2,162 | 1,882 | 1,425 | 1,230 | 1,195 | 964 | 903 | -1259 |
|  | MOEXIPRIL HCL/HCTZ | 13 - 17 | 1 |  |  |  |  |  |  |  |
|  | QUINAPRIL HCL/HCTZ | 3 - 7 |  | 1 |  |  |  |  |  |  |
|  | QUINAPRIL HCL/HCTZ | 8 - 12 | 0 |  |  | 1 |  |  |  |  |
|  | QUINAPRIL HCL/HCTZ | 13 - 17 | 0 | 3 | 2 | 1 |  |  |  |  |
| ALPHA BLOCKERS, ALONE, COMB | DOXAZOSIN MESY | 3 - 7 | 720 | 664 | 482 | 461 | 445 | 395 | 319 | -400 |
|  | DOXAZOSIN MESY | 8 - 12 | 956 | 872 | 750 | 616 | 621 | 629 | 672 | -283 |
|  | DOXAZOSIN MESY | 13 - 17 | 763 | 845 | 788 | 764 | 803 | 829 | 793 | 30 |
|  | PHENOXYBENZAMINE | 3 - 7 | 1 | 3 | 1 | 2 | 1 |  |  |  |
|  | PHENOXYBENZAMINE | 8 - 12 | 5 | 6 | 1 | 3 | 5 | 3 | 8 | 3 |
|  | PHENOXYBENZAMINE | 13 - 17 | 17 | 15 | 11 | 14 | 6 | 9 | 7 | -10 |
|  | PRAZOSIN HCL | 3 - 7 | 1,428 | 1,615 | 1,673 | 1,609 | 1,506 | 1,437 | 1,385 | -43 |
|  | PRAZOSIN HCL | 8 - 12 | 5,291 | 6,379 | 6,870 | 7,580 | 7,975 | 7,852 | 7,661 | 2370 |
|  | PRAZOSIN HCL | 13 - 17 | 24,983 | 28,529 | 31,470 | 34,649 | 39,420 | 40,354 | 37,469 | 12486 |
|  | TERAZOSIN HCL | 3 - 7 | 382 | 432 | 426 | 459 | 551 | 545 | 467 | 85 |
|  | TERAZOSIN HCL | 8 - 12 | 506 | 542 | 561 | 516 | 597 | 705 | 711 | 205 |
|  | TERAZOSIN HCL | 13 - 17 | 378 | 437 | 352 | 378 | 414 | 404 | 438 | 60 |
| ALPHA-BETA BLOCKER | CARVEDILOL | 3 - 7 | 560 | 538 | 333 | 347 | 321 | 325 | 298 | -262 |
|  | CARVEDILOL | 8 - 12 | 1,022 | 963 | 774 | 836 | 738 | 818 | 761 | -261 |
|  | CARVEDILOL | 13 - 17 | 2,254 | 2,091 | 1,799 | 1,821 | 1,820 | 1,841 | 1,766 | -488 |
|  | CARVEDILOL PHOSPH | 3 - 7 |  | 1 |  |  |  | 2 | 1 |  |
|  | CARVEDILOL PHOSPH | 8 - 12 | 2 |  | 1 | 2 | 3 | 3 | 4 | 3 |
|  | CARVEDILOL PHOSPH | 13 - 17 | 7 | 16 | 16 | 13 | 8 | 20 | 18 | 11 |
|  | LABETALOL HCL | 3 - 7 | 123 | 136 | 109 | 123 | 99 | 108 | 95 | -28 |
|  | LABETALOL HCL | 8 - 12 | 414 | 361 | 343 | 297 | 370 | 371 | 388 | -26 |
|  | LABETALOL HCL | 13 - 17 | 1,780 | 1,792 | 1,677 | 1,565 | 1,607 | 1,728 | 1,868 | 88 |
| ANGIO II ANTAG, ALONE | CANDESARTAN CIL | 3 - 7 | 6 | 8 | 19 | 20 | 25 | 17 | 14 | 8 |
|  | CANDESARTAN CIL | 8 - 12 | 33 | 33 | 46 | 62 | 53 | 66 | 68 | 35 |
|  | CANDESARTAN CIL | 13 - 17 | 185 | 216 | 263 | 228 | 193 | 253 | 308 | 123 |
|  | EPROSARTAN MESY | 13 - 17 |  | 1 |  |  |  |  |  |  |
|  | IRBESARTAN | 3 - 7 | 48 | 73 | 76 | 55 | 47 | 54 | 74 | 26 |
|  | IRBESARTAN | 8 - 12 | 79 | 94 | 104 | 141 | 187 | 215 | 231 | 153 |
|  | IRBESARTAN | 13 - 17 | 176 | 222 | 209 | 272 | 326 | 406 | 474 | 297 |
|  | LOSARTAN POT | 3 - 7 | 889 | 945 | 661 | 662 | 545 | 627 | 608 | -281 |
|  | LOSARTAN POT | 8 - 12 | 2,926 | 2,861 | 2,370 | 2,417 | 2,359 | 2,441 | 2,669 | -258 |
|  | LOSARTAN POT | 13 - 17 | 6,620 | 6,861 | 6,165 | 6,194 | 6,834 | 7,671 | 8,026 | 1407 |
|  | OLMESARTAN MEDOX | 3 - 7 | 3 | 12 | 32 | 11 | 8 | 11 | 7 | 4 |
|  | OLMESARTAN MEDOX | 8 - 12 | 10 | 7 | 31 | 29 | 33 | 29 | 48 | 37 |
|  | OLMESARTAN MEDOX | 13 - 17 | 37 | 80 | 100 | 132 | 170 | 187 | 234 | 197 |
|  | TELMISARTAN | 3 - 7 | 123 | 198 | 308 | 414 | 541 | 481 | 596 | 474 |
|  | TELMISARTAN | 8 - 12 | 199 | 486 | 830 | 1,048 | 1,357 | 1,639 | 1,508 | 1309 |
|  | TELMISARTAN | 13 - 17 | 141 | 317 | 321 | 445 | 620 | 769 | 898 | 757 |
|  | VALSARTAN | 3 - 7 | 21 | 29 | 41 | 38 | 11 | 12 | 19 | -2 |
|  | VALSARTAN | 8 - 12 | 71 | 95 | 38 | 56 | 51 | 88 | 89 | 19 |
|  | VALSARTAN | 13 - 17 | 386 | 339 | 158 | 217 | 241 | 371 | 454 | 68 |
| ANGIO II ANTAG,WITH CCB | AMLODIP BES/OLMESAR | 3 - 7 | 1 | 3 | 5 | 4 | 0 | 0 |  |  |
|  | AMLODIP BES/OLMESAR | 8 - 12 | 0 | 5 | 1 | 7 | 2 | 2 | 2 | 2 |
|  | AMLODIP BES/OLMESAR | 13 - 17 | 16 | 17 | 14 | 11 | 8 | 14 | 29 | 14 |
|  | AMLODIP BES/VALSAR | 3 - 7 | 3 | 3 | 16 | 9 |  | 5 |  |  |
|  | AMLODIP BES/VALSAR | 8 - 12 | 9 | 2 | 3 | 3 | 3 | 5 | 4 | -5 |
|  | AMLODIP BES/VALSAR | 13 - 17 | 40 | 40 | 28 | 28 | 75 | 66 | 82 | 42 |
|  | TELMISARTAN/AMLODIP | 8 - 12 |  |  |  | 1 | 2 |  | 1 |  |
|  | TELMISARTAN/AMLODIP | 13 - 17 |  | 1 |  |  | 5 | 14 | 13 |  |
| ANGIO II ANTAG, WITH CCB/DIURET | AMLODIP/VALS/HCTZ | 3 - 7 | 1 | 1 | 2 | 0 |  |  |  |  |
|  | AMLODIP/VALS/HCTZ | 8 - 12 | 4 | 4 | 0 | 2 | 2 |  | 1 | -3 |
|  | AMLODIP/VALS/HCTZ | 13 - 17 | 29 | 18 | 9 | 21 | 9 | 4 | 14 | -15 |
|  | OLMSRTN/AMLDPN/HCTZ | 3 - 7 | 0 | 1 | 2 | 1 |  |  | 1 | 1 |
|  | OLMSRTN/AMLDPN/HCTZ | 8 - 12 | 0 | 1 | 1 | 0 |  | 1 | 1 | 1 |
|  | OLMSRTN/AMLDPN/HCTZ | 13 - 17 | 2 | 4 | 2 | 2 |  | 2 | 3 | 1 |
| ANGIO II ANTAG, WITH CCB/DIURETICS | CANDESARTAN-HCTZ | 3 - 7 |  |  |  |  | 1 |  |  |  |
|  | CANDESARTAN-HCTZ | 8 - 12 | 0 | 2 | 0 |  |  | 0 | 2 | 2 |
|  | CANDESARTAN-HCTZ | 13 - 17 | 4 | 3 | 7 | 16 | 7 | 6 | 14 | 11 |
|  | IRBESARTAN/HCTZ | 3 - 7 | 0 | 2 | 7 | 3 | 4 | 2 |  |  |
|  | IRBESARTAN/HCTZ | 8 - 12 | 1 | 1 | 2 | 1 | 5 | 4 | 2 | 1 |
|  | IRBESARTAN/HCTZ | 13 - 17 | 16 | 10 | 7 | 10 | 14 | 14 | 16 | 0 |
|  | LOSARTAN POT/HCTZ | 3 - 7 | 11 | 15 | 22 | 13 | 13 | 18 | 6 | -5 |
|  | LOSARTAN POT/HCTZ | 8 - 12 | 72 | 80 | 49 | 33 | 44 | 43 | 36 | -36 |
|  | LOSARTAN POT/HCTZ | 13 - 17 | 548 | 607 | 375 | 299 | 343 | 357 | 402 | -146 |
|  | OLMESARTAN/HCTZ | 3 - 7 | 3 | 6 | 30 | 9 | 1 | 2 | 4 | 1 |
|  | OLMESARTAN/HCTZ | 8 - 12 | 6 | 7 | 7 | 3 | 9 | 3 | 4 | -3 |
|  | OLMESARTAN/HCTZ | 13 - 17 | 15 | 14 | 22 | 30 | 25 | 26 | 35 | 20 |
|  | TELMISARTAN/HCTZ | 3 - 7 | 3 | 5 | 6 | 3 | 2 |  |  |  |
|  | TELMISARTAN/HCTZ | 8 - 12 | 1 | 2 | 0 | 1 | 0 | 3 | 3 | 2 |
|  | TELMISARTAN/HCTZ | 13 - 17 | 9 | 2 | 4 | 9 | 6 | 8 | 4 | -5 |
|  | VALSARTAN-HCTZ | 3 - 7 | 8 | 15 | 6 | 10 | 2 | 5 | 2 | -6 |
|  | VALSARTAN-HCTZ | 8 - 12 | 9 | 17 | 6 | 6 | 17 | 7 | 9 | 0 |
|  | VALSARTAN-HCTZ | 13 - 17 | 88 | 92 | 38 | 49 | 70 | 82 | 87 | -1 |
| BETA BLOCKERS | ACEBUTOLOL HCL | 3 - 7 | 6 | 4 |  |  | 3 | 3 | 1 | -5 |
|  | ACEBUTOLOL HCL | 8 - 12 | 22 | 16 | 13 | 9 | 10 | 7 | 10 | -12 |
|  | ACEBUTOLOL HCL | 13 - 17 | 34 | 53 | 33 | 30 | 23 | 17 | 18 | -16 |
|  | ATENOLOL | 3 - 7 | 2,996 | 2,712 | 2,210 | 2,364 | 2,536 | 2,604 | 2,599 | -397 |
|  | ATENOLOL | 8 - 12 | 6,197 | 5,615 | 5,584 | 5,348 | 5,505 | 5,426 | 5,379 | -818 |
|  | ATENOLOL | 13 - 17 | 14,311 | 12,754 | 12,240 | 11,431 | 12,287 | 12,391 | 11,952 | -2358 |
|  | BETAXOLOL HCL | 3 - 7 | 8 | 4 | 4 | 4 | 1 | 3 | 2 | -6 |
|  | BETAXOLOL HCL | 8 - 12 | 30 | 29 | 15 | 15 | 13 | 10 | 7 | -23 |
|  | BETAXOLOL HCL | 13 - 17 | 143 | 117 | 122 | 77 | 84 | 97 | 82 | -61 |
|  | BISOPROLOL FUM | 3 - 7 | 12 | 15 | 22 | 20 | 23 | 20 | 27 | 15 |
|  | BISOPROLOL FUM | 8 - 12 | 53 | 54 | 46 | 63 | 87 | 84 | 65 | 12 |
|  | BISOPROLOL FUM | 13 - 17 | 320 | 278 | 210 | 205 | 279 | 323 | 345 | 25 |
|  | METOPROLOL SUCCIN | 3 - 7 | 236 | 261 | 130 | 131 | 127 | 155 | 138 | -98 |
|  | METOPROLOL SUCCIN | 8 - 12 | 1,427 | 1,521 | 1,156 | 1,178 | 1,045 | 1,153 | 1,363 | -64 |
|  | METOPROLOL SUCCIN | 13 - 17 | 6,794 | 6,771 | 6,123 | 6,092 | 6,667 | 7,337 | 7,669 | 875 |
|  | METOPROLOL TART | 3 - 7 | 281 | 303 | 189 | 202 | 157 | 186 | 162 | -119 |
|  | METOPROLOL TART | 8 - 12 | 1,054 | 1,010 | 744 | 701 | 589 | 585 | 631 | -423 |
|  | METOPROLOL TART | 13 - 17 | 3,995 | 3,854 | 3,278 | 2,902 | 3,011 | 3,019 | 2,818 | -1177 |
|  | NADOLOL | 3 - 7 | 644 | 680 | 742 | 719 | 757 | 759 | 815 | 170 |
|  | NADOLOL | 8 - 12 | 1,856 | 1,748 | 1,857 | 1,699 | 1,804 | 2,043 | 2,075 | 219 |
|  | NADOLOL | 13 - 17 | 3,671 | 3,649 | 3,587 | 3,326 | 3,348 | 3,561 | 3,686 | 15 |
|  | NEBIVOLOL HCL | 3 - 7 |  |  |  |  | 5 | 11 | 6 |  |
|  | NEBIVOLOL HCL | 8 - 12 |  |  |  |  | 15 | 33 | 27 |  |
|  | NEBIVOLOL HCL | 13 - 17 |  |  |  |  | 60 | 197 | 203 |  |
|  | PINDOLOL | 3 - 7 | 9 | 6 | 5 | 6 | 1 | 1 |  |  |
|  | PINDOLOL | 8 - 12 | 30 | 17 | 25 | 11 | 6 | 11 | 5 | -25 |
|  | PINDOLOL | 13 - 17 | 114 | 127 | 149 | 100 | 87 | 82 | 112 | -1 |
|  | PROPRANOLOL HCL | 3 - 7 | 4,412 | 4,386 | 4,367 | 4,081 | 4,290 | 4,382 | 4,329 | -83 |
|  | PROPRANOLOL HCL | 8 - 12 | 11,655 | 11,483 | 11,554 | 10,103 | 11,173 | 12,139 | 12,719 | 1064 |
|  | PROPRANOLOL HCL | 13 - 17 | 48,858 | 52,309 | 56,145 | 51,208 | 62,797 | 73,780 | 77,663 | 28805 |
|  | TIMOLOL MAL | 8 - 12 | 3 | 1 | 2 | 1 | 1 |  |  |  |
|  | TIMOLOL MAL | 13 - 17 | 35 | 37 | 8 | 9 | 25 | 6 | 6 | -29 |
| BETA/ ALPHA-BETA BLOCKER WITH DIURETICS | ATENOLOL/CHLORTHAL | 3 - 7 | 8 | 8 | 4 | 1 |  | 1 |  |  |
|  | ATENOLOL/CHLORTHAL | 8 - 12 | 9 | 9 | 8 | 5 | 7 | 2 | 1 | -8 |
|  | ATENOLOL/CHLORTHAL | 13 - 17 | 110 | 131 | 106 | 77 | 65 | 39 | 41 | -69 |
|  | BISOPROLOL FUM/HCTZ | 3 - 7 | 4 | 5 | 4 | 4 | 1 |  | 1 | -3 |
|  | BISOPROLOL FUM/HCTZ | 8 - 12 | 21 | 13 | 8 | 4 | 9 | 1 | 1 | -20 |
|  | BISOPROLOL FUM/HCTZ | 13 - 17 | 84 | 69 | 46 | 38 | 29 | 24 | 11 | -73 |
|  | METOPROLOL/HCTZ | 3 - 7 | 0 | 2 |  |  |  |  |  |  |
|  | METOPROLOL/HCTZ | 8 - 12 | 0 |  | 1 | 2 |  |  |  |  |
|  | METOPROLOL/HCTZ | 13 - 17 | 10 | 14 | 5 | 7 | 8 | 7 | 2 | -8 |
|  | NADOLOL/BENDROFLUM | 13 - 17 | 2 |  |  |  |  |  |  |  |
|  | PROPRANOLOL/HCTZ | 8 - 12 |  | 1 |  |  |  |  |  |  |
|  | PROPRANOLOL/HCTZ | 13 - 17 | 1 | 6 | 1 | 2 |  |  |  |  |
| CALCIUM BLOCKERS | AMLODIPINE BESY | 3 - 7 | 2,753 | 2,905 | 2,268 | 2,237 | 2,120 | 2,151 | 2,125 | -628 |
|  | AMLODIPINE BESY | 8 - 12 | 7,149 | 7,456 | 6,974 | 6,900 | 7,017 | 7,170 | 7,077 | -72 |
|  | AMLODIPINE BESY | 13 - 17 | 17,233 | 17,820 | 15,926 | 15,667 | 17,406 | 17,368 | 17,176 | -57 |
|  | DILTIAZEM 24HR | 3 - 7 | 6 | 5 | 1 |  |  |  |  |  |
|  | DILTIAZEM 24HR | 8 - 12 | 7 | 1 | 3 | 1 |  |  |  |  |
|  | DILTIAZEM 24HR | 13 - 17 | 9 | 6 | 6 | 5 | 3 |  |  |  |
|  | DILTIAZEM HCL | 3 - 7 | 520 | 450 | 271 | 328 | 257 | 257 | 213 | -307 |
|  | DILTIAZEM HCL | 8 - 12 | 741 | 786 | 591 | 633 | 566 | 515 | 453 | -288 |
|  | DILTIAZEM HCL | 13 - 17 | 922 | 863 | 649 | 648 | 646 | 616 | 519 | -403 |
|  | DILTIAZEM SR | 3 - 7 | 11 | 18 | 15 | 22 | 13 | 15 | 9 | -2 |
|  | DILTIAZEM SR | 8 - 12 | 26 | 36 | 21 | 16 | 40 | 35 | 38 | 12 |
|  | DILTIAZEM SR | 13 - 17 | 78 | 56 | 40 | 47 | 33 | 43 | 50 | -28 |
|  | DILTIAZEM XR | 3 - 7 | 37 | 14 | 1 | 6 | 25 | 46 | 42 | 6 |
|  | DILTIAZEM XR | 8 - 12 | 59 | 35 | 9 | 11 | 42 | 58 | 64 | 5 |
|  | DILTIAZEM XR | 13 - 17 | 55 | 29 | 11 |  | 8 | 27 | 55 | 0 |
|  | FELODIPINE ER | 3 - 7 | 2 | 2 | 3 | 2 | 5 |  |  |  |
|  | FELODIPINE ER | 8 - 12 | 7 | 14 | 14 | 7 | 4 | 4 | 5 | -2 |
|  | FELODIPINE ER | 13 - 17 | 31 | 35 | 38 | 38 | 29 | 24 | 14 | -17 |
|  | ISRADIPINE | 3 - 7 | 45 | 38 | 25 | 27 | 29 | 25 | 25 | -19 |
|  | ISRADIPINE | 8 - 12 | 112 | 125 | 122 | 92 | 104 | 104 | 97 | -15 |
|  | ISRADIPINE | 13 - 17 | 197 | 237 | 189 | 225 | 241 | 213 | 192 | -5 |
|  | MATZIM LA | 3 - 7 | 1 | 1 |  |  |  |  |  |  |
|  | MATZIM LA | 8 - 12 | 3 | 2 | 1 |  |  |  | 4 | 0 |
|  | MATZIM LA | 13 - 17 | 5 | 5 | 5 | 3 | 3 | 2 | 4 | -2 |
|  | NICARDIPINE HCL | 13 - 17 | 3 | 5 | 8 | 4 |  |  |  |  |
|  | NIFEDIPINE | 3 - 7 | 19 | 20 | 10 | 17 | 14 | 6 | 9 | -11 |
|  | NIFEDIPINE | 8 - 12 | 66 | 81 | 42 | 56 | 48 | 19 | 31 | -35 |
|  | NIFEDIPINE | 13 - 17 | 773 | 636 | 542 | 441 | 460 | 376 | 340 | -433 |
|  | NIFEDIPINE ER | 3 - 7 | 36 | 35 | 17 | 10 | 13 | 17 | 15 | -21 |
|  | NIFEDIPINE ER | 8 - 12 | 243 | 270 | 219 | 184 | 202 | 243 | 244 | 1 |
|  | NIFEDIPINE ER | 13 - 17 | 2,112 | 2,228 | 2,165 | 2,138 | 2,280 | 2,256 | 2,467 | 355 |
|  | NIMODIPINE | 3 - 7 | 0 |  |  | 2 |  |  | 0 | 0 |
|  | NIMODIPINE | 8 - 12 | 8 | 8 | 3 | 4 | 6 | 36 | 15 | 6 |
|  | NIMODIPINE | 13 - 17 | 14 | 30 | 34 | 19 | 17 | 9 | 9 | -5 |
|  | NISOLDIPINE | 3 - 7 | 1 | 3 |  |  |  |  |  |  |
|  | NISOLDIPINE | 8 - 12 | 0 |  |  |  |  |  |  |  |
|  | NISOLDIPINE | 13 - 17 | 0 |  |  |  |  | 1 | 1 | 1 |
|  | VERAPAMIL HCL | 3 - 7 | 93 | 77 | 68 | 71 | 55 | 47 | 58 | -35 |
|  | VERAPAMIL HCL | 8 - 12 | 533 | 457 | 400 | 318 | 293 | 279 | 293 | -240 |
|  | VERAPAMIL HCL | 13 - 17 | 2,122 | 2,035 | 1,882 | 1,347 | 1,294 | 1,227 | 1,105 | -1017 |
|  | VERAPAMIL SR | 3 - 7 | 32 | 35 | 11 | 14 | 25 | 15 | 19 | -13 |
|  | VERAPAMIL SR | 8 - 12 | 460 | 323 | 225 | 197 | 177 | 204 | 232 | -228 |
|  | VERAPAMIL SR | 13 - 17 | 2,394 | 2,018 | 1,772 | 1,434 | 1,410 | 1,258 | 1,322 | -1072 |
|  | VERAPAMIL SR PM | 3 - 7 | 2 | 1 | 4 | 1 | 4 | 2 | 3 | 1 |
|  | VERAPAMIL SR PM | 8 - 12 | 31 | 33 | 34 | 10 | 13 | 16 | 19 | -12 |
|  | VERAPAMIL SR PM | 13 - 17 | 208 | 197 | 159 | 118 | 98 | 77 | 62 | -145 |
| CENTRAL ACTING AGENT, ALONE, COMB | CLONIDINE | 3 - 7 | 670 | 797 | 787 | 725 | 684 | 669 | 996 | 326 |
|  | CLONIDINE | 8 - 12 | 1,102 | 1,113 | 1,212 | 1,166 | 1,206 | 1,064 | 1,584 | 483 |
|  | CLONIDINE | 13 - 17 | 1,051 | 1,064 | 1,125 | 1,133 | 1,113 | 1,056 | 1,267 | 216 |
|  | CLONIDINE HCL | 3 - 7 | 118,669 | 117,456 | 117,705 | 118,111 | 116,595 | 114,732 | 116,875 | -1794 |
|  | CLONIDINE HCL | 8 - 12 | 291,498 | 289,434 | 293,711 | 292,539 | 293,105 | 293,718 | 288,218 | -3280 |
|  | CLONIDINE HCL | 13 - 17 | 214,039 | 221,901 | 232,226 | 242,660 | 263,755 | 270,967 | 265,816 | 51778 |
|  | GUANFACINE HCL | 3 - 7 | 89,453 | 90,472 | 87,170 | 85,090 | 85,864 | 91,550 | 97,803 | 8349 |
|  | GUANFACINE HCL | 8 - 12 | 169,426 | 163,519 | 154,211 | 143,159 | 140,279 | 143,998 | 144,904 | -24522 |
|  | GUANFACINE HCL | 13 - 17 | 93,202 | 93,662 | 87,709 | 84,475 | 88,575 | 88,643 | 86,541 | -6661 |
|  | METHYLDOPA | 3 - 7 | 7 | 10 |  | 1 |  |  |  |  |
|  | METHYLDOPA | 8 - 12 | 2 | 4 |  | 2 |  |  |  |  |
|  | METHYLDOPA | 13 - 17 | 94 | 75 | 23 | 15 | 1 |  | 1 | -93 |
|  | METHYLDOPA/HCTZ | 8 - 12 |  |  | 1 |  |  |  |  |  |
| DIRECT RENIN INHIB, ALONE | ALISKIREN HEMIFUM | 3 - 7 |  |  | 1 |  |  |  |  |  |
|  | ALISKIREN HEMIFUM | 8 - 12 |  |  |  | 1 |  |  | 1 |  |
|  | ALISKIREN HEMIFUM | 13 - 17 |  |  | 1 | 1 | 2 | 2 | 4 |  |
| DIURETICS, COMB | AMILORIDE HCL/HCTZ | 3 - 7 | 9 | 5 | 4 | 6 | 2 | 1 |  |  |
|  | AMILORIDE HCL/HCTZ | 8 - 12 | 28 | 32 | 19 | 13 | 16 | 20 | 18 | -11 |
|  | AMILORIDE HCL/HCTZ | 13 - 17 | 57 | 58 | 28 | 28 | 19 | 24 | 27 | -30 |
|  | SPIRONOLACTONE/HCT | 3 - 7 | 114 | 117 | 96 | 96 | 123 | 79 | 78 | -37 |
|  | SPIRONOLACTONE/HCT | 8 - 12 | 142 | 146 | 107 | 114 | 116 | 105 | 75 | -67 |
|  | SPIRONOLACTONE/HCT | 13 - 17 | 198 | 179 | 165 | 148 | 164 | 155 | 130 | -68 |
|  | TRIAMTERENE/HCTZ | 3 - 7 | 25 | 26 | 13 | 13 | 4 | 1 | 5 | -19 |
|  | TRIAMTERENE/HCTZ | 8 - 12 | 20 | 27 | 57 | 62 | 14 | 7 | 16 | -4 |
|  | TRIAMTERENE/HCTZ | 13 - 17 | 393 | 323 | 264 | 177 | 233 | 174 | 121 | -271 |
| DIURETICS,LOOP | BUMETANIDE | 3 - 7 | 203 | 209 | 185 | 217 | 235 | 186 | 201 | -2 |
|  | BUMETANIDE | 8 - 12 | 107 | 121 | 146 | 148 | 165 | 150 | 147 | 40 |
|  | BUMETANIDE | 13 - 17 | 150 | 166 | 138 | 166 | 166 | 170 | 174 | 24 |
|  | ETHACRYNIC ACID | 3 - 7 |  | 1 |  |  |  | 2 | 12 |  |
|  | ETHACRYNIC ACID | 8 - 12 | 3 | 2 |  | 2 |  |  |  |  |
|  | ETHACRYNIC ACID | 13 - 17 |  | 1 | 1 | 2 | 13 | 3 | 2 |  |
|  | FUROSEMIDE | 3 - 7 | 8,346 | 7,704 | 6,812 | 6,214 | 6,050 | 5,965 | 5,730 | -2617 |
|  | FUROSEMIDE | 8 - 12 | 8,487 | 8,669 | 7,432 | 7,229 | 6,324 | 6,257 | 5,633 | -2854 |
|  | FUROSEMIDE | 13 - 17 | 9,912 | 9,820 | 6,932 | 6,681 | 6,601 | 6,305 | 6,428 | -3484 |
|  | TORSEMIDE | 3 - 7 | 104 | 138 | 111 | 96 | 102 | 96 | 104 | 0 |
|  | TORSEMIDE | 8 - 12 | 304 | 390 | 407 | 428 | 388 | 302 | 269 | -35 |
|  | TORSEMIDE | 13 - 17 | 245 | 241 | 196 | 210 | 218 | 199 | 202 | -43 |
| DIURETICS,POT SPARING | AMILORIDE HCL | 3 - 7 | 80 | 88 | 87 | 75 | 61 | 60 | 58 | -22 |
|  | AMILORIDE HCL | 8 - 12 | 178 | 172 | 172 | 169 | 192 | 174 | 182 | 4 |
|  | AMILORIDE HCL | 13 - 17 | 344 | 348 | 321 | 326 | 314 | 307 | 334 | -10 |
|  | SPIRONOLACTONE | 3 - 7 | 1,867 | 1,726 | 1,390 | 1,383 | 1,379 | 1,344 | 1,475 | -393 |
|  | SPIRONOLACTONE | 8 - 12 | 4,116 | 4,604 | 4,372 | 4,421 | 4,734 | 4,858 | 4,646 | 530 |
|  | SPIRONOLACTONE | 13 - 17 | 39,756 | 47,544 | 55,461 | 64,220 | 79,236 | 85,813 | 92,303 | 52547 |
|  | TRIAMTERENE | 3 - 7 |  |  |  |  |  | 1 |  |  |
|  | TRIAMTERENE | 8 - 12 |  |  |  |  | 2 |  | 1 |  |
|  | TRIAMTERENE | 13 - 17 |  |  | 2 | 5 | 4 | 8 | 8 |  |
| DIURETICS, THIAZIDE & RELATED | CHLOROTHIAZIDE | 3 - 7 | 173 | 123 | 106 | 22 |  |  |  |  |
|  | CHLOROTHIAZIDE | 8 - 12 | 222 | 203 | 178 | 51 |  |  |  |  |
|  | CHLOROTHIAZIDE | 13 - 17 | 292 | 314 | 279 | 80 | 2 | 2 |  |  |
|  | CHLORTHALIDONE | 3 - 7 | 67 | 69 | 34 | 38 | 33 | 28 | 21 | -45 |
|  | CHLORTHALIDONE | 8 - 12 | 186 | 188 | 159 | 146 | 130 | 126 | 112 | -74 |
|  | CHLORTHALIDONE | 13 - 17 | 641 | 583 | 556 | 551 | 511 | 498 | 393 | -248 |
|  | HYDROCHLOROTHIAZIDE | 3 - 7 | 698 | 706 | 463 | 612 | 524 | 546 | 621 | -77 |
|  | HYDROCHLOROTHIAZIDE | 8 - 12 | 1,921 | 1,788 | 1,457 | 1,545 | 1,420 | 1,386 | 1,271 | -651 |
|  | HYDROCHLOROTHIAZIDE | 13 - 17 | 6,157 | 5,867 | 4,959 | 4,696 | 4,580 | 3,966 | 3,596 | -2561 |
|  | INDAPAMIDE | 3 - 7 | 6 | 2 | 0 | 1 | 1 | 0 |  |  |
|  | INDAPAMIDE | 8 - 12 | 9 | 10 | 4 | 7 |  | 1 | 3 | -6 |
|  | INDAPAMIDE | 13 - 17 | 38 | 35 | 20 | 12 | 12 | 15 | 13 | -25 |
|  | METHYCLOTHIAZIDE | 3 - 7 | 1 | 1 |  |  |  |  |  |  |
|  | METHYCLOTHIAZIDE | 8 - 12 |  |  | 1 |  |  |  |  |  |
|  | METHYCLOTHIAZIDE | 13 - 17 |  |  | 1 |  |  |  |  |  |
|  | METOLAZONE | 3 - 7 | 38 | 50 | 32 | 30 | 22 | 29 | 40 | 2 |
|  | METOLAZONE | 8 - 12 | 90 | 100 | 59 | 58 | 43 | 54 | 51 | -39 |
|  | METOLAZONE | 13 - 17 | 150 | 133 | 96 | 91 | 60 | 83 | 95 | -56 |
| SEL ALDOSTERONE RECPT ANTAG | EPLERENONE | 3 - 7 | 3 | 8 | 11 | 5 | 11 | 3 | 5 | 1 |
|  | EPLERENONE | 8 - 12 | 51 | 82 | 89 | 102 | 104 | 141 | 151 | 100 |
|  | EPLERENONE | 13 - 17 | 174 | 201 | 238 | 292 | 397 | 518 | 521 | 347 |
| VASC/ANTIHYPERLIPIDEMIC COMB | AMLODIP BES/ATORVAST | 3 - 7 |  | 1 | 2 | 1 |  |  |  |  |
|  | AMLODIP BES/ATORVAST | 8 - 12 | 2 | 1 |  | 1 |  |  |  |  |
|  | AMLODIP BES/ATORVAST | 13 - 17 | 14 | 17 | 6 | 2 | 2 | 2 | 2 | -12 |
| VASCULAR AGENTS, OTHER | HYDRALAZINE HCL | 3 - 7 | 91 | 121 | 98 | 80 | 73 | 68 | 79 | -12 |
|  | HYDRALAZINE HCL | 8 - 12 | 239 | 233 | 234 | 190 | 182 | 172 | 159 | -80 |
|  | HYDRALAZINE HCL | 13 - 17 | 510 | 519 | 468 | 429 | 409 | 434 | 445 | -65 |
|  | METYROSINE | 13 - 17 |  |  |  | 1 | 2 | 3 | 5 |  |
|  | MINOXIDIL | 3 - 7 | 28 | 24 | 31 | 29 | 39 | 131 | 299 | 270 |
|  | MINOXIDIL | 8 - 12 | 66 | 43 | 56 | 65 | 132 | 369 | 668 | 603 |
|  | MINOXIDIL | 13 - 17 | 112 | 113 | 161 | 243 | 489 | 1,372 | 2,706 | 2594 |

Abbreviations: ; ACE, angiotensin-converting enzyme; ACT, acting; AGT, agent; ANTAG, antagonist; BES(Y), besylate; CCB, calcium channel blocker; CIL, cilexitil; COMB, combination; Diff, difference; ER, extended release; FUM, fumarate; HCL, hydrochloride; HCTZ, hydrochlorothiazide; LS, long-acting; MAL, maleate; MEDOX, medoxomil; MESY, mesylate; OLMESAR, olmesartan; POT, potassium; PM, post meridem; RECPT, receptor; SEL, selective; SOD, sodium; SR, sustained release; VALSAR, valsartan; VASC, vascular; XR, extended release

^a^ This Appendix Tablehows the annual counts of unique number of individuals aged 0 to 17 years with BP-lowering prescription fills each calendar year during 2017–2023. Empty cells denote count to be equal to 0.

^b^ This column shows difference in unique number of individuals with BP-lowering prescription fills in year 2023 in comparison to year 2017.

**Appendix Table5:** Annual total number of unique individuals aged 3 to 17 years with prescription fills for generic BP-lowering medications that had greatest increase (>500) in number of individuals filling prescriptions during 2017–2023, IQVIA Total Patient Tracker, 2017–2023^a^

| **Drug class** | **Product brand** | **Age group** | **2017** | **2018** | **2019** | **2020** | **2021** | **2022** | **2023** | **Diff^b^** |
| --- | --- | --- | --- | --- | --- | --- | --- | --- | --- | --- |
| ACE INHIBITOR, ALONE | ENALAPRIL MAL | 3 - 7 | 4,866 | 3,896 | 3,128 | 2,866 | 5,004 | 6,800 | 6,694 | 1828 |
| ALPHA BLOCKERS, ALONE, COMB | PRAZOSIN HCL | 8 - 12 | 5,291 | 6,379 | 6,870 | 7,580 | 7,975 | 7,852 | 7,661 | 2370 |
|  | PRAZOSIN HCL | 13 - 17 | 24,983 | 28,529 | 31,470 | 34,649 | 39,420 | 40,354 | 37,469 | 12486 |
| ANGIO II ANTAG, ALONE | LOSARTAN POT | 13 - 17 | 6,620 | 6,861 | 6,165 | 6,194 | 6,834 | 7,671 | 8,026 | 1407 |
|  | TELMISARTAN | 8 - 12 | 199 | 486 | 830 | 1,048 | 1,357 | 1,639 | 1,508 | 1309 |
|  | TELMISARTAN | 13 - 17 | 141 | 317 | 321 | 445 | 620 | 769 | 898 | 757 |
| BETA BLOCKERS | METOPROLOL SUCCIN | 13 - 17 | 6,794 | 6,771 | 6,123 | 6,092 | 6,667 | 7,337 | 7,669 | 875 |
|  | PROPRANOLOL HCL | 8 - 12 | 11,655 | 11,483 | 11,554 | 10,103 | 11,173 | 12,139 | 12,719 | 1064 |
|  | PROPRANOLOL HCL | 13 - 17 | 48,858 | 52,309 | 56,145 | 51,208 | 62,797 | 73,780 | 77,663 | 28805 |
| CENTRAL ACT AGT, ALONE, COMB | CLONIDINE HCL | 13 - 17 | 214,039 | 221,901 | 232,226 | 242,660 | 263,755 | 270,967 | 265,816 | 51778 |
|  | GUANFACINE HCL | 3 - 7 | 89,453 | 90,472 | 87,170 | 85,090 | 85,864 | 91,550 | 97,803 | 8349 |
| DIURETICS, POT SPARING | SPIRONOLACTONE | 8 - 12 | 4,116 | 4,604 | 4,372 | 4,421 | 4,734 | 4,858 | 4,646 | 530 |
|  | SPIRONOLACTONE | 13 - 17 | 39,756 | 47,544 | 55,461 | 64,220 | 79,236 | 85,813 | 92,303 | 52547 |
| VASCULAR AGENTS, OTH | MINOXIDIL | 8 - 12 | 66 | 43 | 56 | 65 | 132 | 369 | 668 | 603 |
|  | MINOXIDIL | 13 - 17 | 112 | 113 | 161 | 243 | 489 | 1,372 | 2,706 | 2594 |

Abbreviations: Diff, difference; HCL, hydrochloride; MAL, maleate; POT, potassium

^a^ This table shows the annual counts of the unique number of individuals aged 3 to 17 years with BP-lowering prescription fills each calendar year during 2017–2023.

^b^ This column shows difference in the unique number of individuals with BP-lowering prescription fills in the year 2023 in comparison to the year 2017.
